# Automated Flood Depth Estimates from Online Traffic Sign Images: Explorations of a Convolutional Neural Network-Based Method

**DOI:** 10.3390/s21165614

**Published:** 2021-08-20

**Authors:** Zhiqing Song, Ye Tuo

**Affiliations:** Department of Civil, Geo and Environmental Engineering, Technical University of Munich, Arcisstrasse 21, 80333 Munich, Germany; zhiqing.song@tum.de

**Keywords:** flood depth, water level, deep learning, instance segmentation, computer vision, flood monitoring

## Abstract

Flood depth monitoring is crucial for flood warning systems and damage control, especially in the event of an urban flood. Existing gauge station data and remote sensing data still has limited spatial and temporal resolution and coverage. Therefore, to expand flood depth data source taking use of online image resources in an efficient manner, an automated, low-cost, and real-time working frame called FloodMask was developed to obtain flood depth from online images containing flooded traffic signs. The method was built on the deep learning framework of Mask R-CNN (regional convolutional neural network), trained by collected and manually annotated traffic sign images. Following further the proposed image processing frame, flood depth data were retrieved more efficiently than manual estimations. As the main results, the flood depth estimates from images (without any mirror reflection and other inference problems) have an average error of 0.11 m, when compared to human visual inspection measurements. This developed method can be further coupled with street CCTV cameras, social media photos, and on-board vehicle cameras to facilitate the development of a smart city with a prompt and efficient flood monitoring system. In future studies, distortion and mirror reflection should be tackled properly to increase the quality of the flood depth estimates.

## 1. Introduction

Flood is a natural disaster that brings major loss to human society and the environment [1]. Various flood models have been established for flood monitoring and forecasting [2,3,4]. However, the state of art of hydrological modeling is that no existing model can forecasts flash flood in a reliable way [2]. Due to the requirement of large data input for models and booming data collection approaches, more efficient and automated data processing ways are in demand. Furthermore, sophisticated large-scale analysis of flooding has been established by the existing precipitation forecast systems, but an accurate and instant platform is in demand for subdivision river areas [5,6].

In flood modeling, the water level is one of the most important parameters for precisely assessing the flood impact and flood forecast, which are obtained by remote sensing but with limitations in water level data sources [7,8]. Cian et al. presents a semiautomatic way for deriving flood depth, using SAR (synthetic aperture radar) imagery and statistical estimation of digital elevation models from LIDAR (light detection and ranging) [9]. A variety of satellite remote sensing data are summarized for river inundation area measurements, from which the inundation area can be estimated and then delineated by many passive and active sensors operating in the visible and microwave range [10,11,12]. However, those methods are short in temporal and spatial resolution as the data is from remote sensing measurements.

Other than remote sensing data, sensors and other hardware are utilized for more data sources to improve the spatial and temporal resolutions of water level data [13,14]. Marin-Perez et al. presented a low-power and long-range communication device, which is a real-time measurement system using on-site sensors [15]. Case studies have been established to determine the water level using a platform integrating unmanned aerial vehicles. Image processing techniques, such as the Canny method, were applied to retrieve the water level, which gave an overall mean error of 0.05 m compared with true values [16]. However, extraneous investment and maintenance efforts of sensor devices, airborne vehicles, and cameras are indispensable to establish such a platform or network [16].

Novel approaches to extract water depth are forthcoming in academic research as artificial intelligence algorithms develop [17]. Event recognition has been improved by deep learning frameworks, which appears to have multimedia contents, including single images, personal photos, videos, and audio recordings [18]. Additionally, Chang et al. summarized various machine learning methods applied in flood forecast modeling, and reported that machine learning methods are the key in developing promising early flood warning systems for urban flood protection [19]. Moreover, Moy de Vitry et al. presented a new scalable flood level trend monitoring approach using 10 surveillance camera systems with a deep convolutional neural network to detect floodwater in surveillance footage and map the flooded area, making it possible to monitor the flood water without on-site camera calibration [20]. However, the high cost of infrastructure and maintenance constrains its massive application to derive precise flood depth data. In the work of Kopp et al., the convolutional neural network was successfully applied with the Mask R-CNN framework to automatedly measure snow depth, which is an important input variable for snow water-generating river models [21]. Similar research has been done based on multiple objects in social media images to obtain flood depth [22]. These studies clearly indicate the high portability of the convolutional neural network in image detection to be applied in many fields in flood monitoring, modeling, and forecasting.

Thus, this study set out to expand the number of limited flood depth data sources using deep learning methods in an efficient and rather accurate manner as an alternative to remote sensing and on-site sensor network. Here, stop signs were utilized as an example type of the traffic signs to develop and evaluate the flood depth extraction method (FloodMask) based on online image resources. The FloodMask is developed based on Mask R-CNN. It was first trained by annotated stop sign images to learn to segment the stop sign out in an image, then images highlighting the masked targets from the collected stop sign images with flood were produced. In the end, flood depth data were estimated based on the local traffic sign dimension. To evaluate the method, manual measurements were obtained by visual observation on raw input traffic sign-containing flood images. The comparison of the automated method with manual measurements completed our objectives of (1) testifying the feasibility of water level extraction with Mask R-CNN; (2) evaluating the precision of FloodMask, as well as post image processing; and (3) investigating the application scenarios of the FloodMask method.

## 2. Materials and Methods

**Figure 1:**
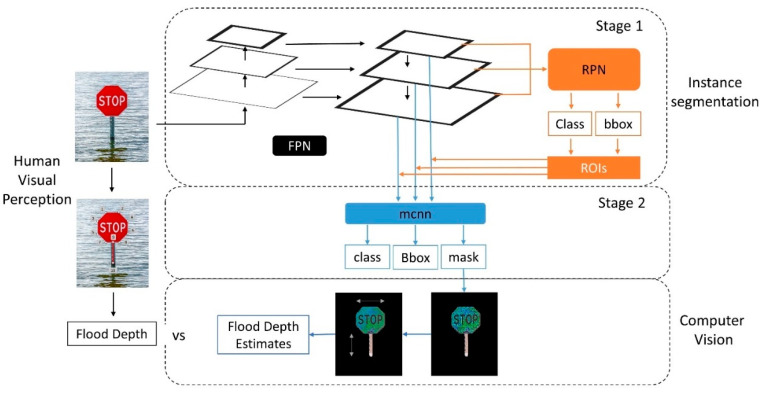
Overall architecture of FloodMask.

The overall architecture of FloodMask is illustrated in Figure 1. Here, three subsections narrate the rationale of the deep learning approach and the post image processing steps are described. In Section 2.1, we demonstrate the 2-step architecture of the deep learning approach, the Mask R-CNN, for the masking and detection of the object in the images. In Section 2.2, assumptions are provided to support the new perspective of flood-water level collection. Thirdly, in Section 2.3, the flood-water level data is extracted from masked images produced by Mask R-CNN, and we present two different methods A and B in this step in FloodMask. Additionally, flood depth data extracted by human visual perception from the same input images for Mask R-CNN are used as references to evaluate the performance of the FloodMask results with the error values. According to the interaction condition between the sign and flood water in each input image, the images can be grouped in three scenarios: (1) regular scenario, where a clear sign could be recognized by visual perception and no problematic scenarios occurred in the image; (2) mirror reflection scenario, where the traffic sign is reflected by the flood water surface partly and the reflection was segmented; and (3) multi-object scenario, where other objects are segmented due to sign modification and detection failure.

### 2.1. Mask R-CNN

Mask R-CNN is a two-stage deep learning framework used for identification of the object contour at the pixel level in an image and is currently widely used in the image detection field [23]. Figure 1 shows the general architecture in this study and illustrates the Mask R-CNN structure with a stop sign image and an output image input with a masked and highlighted sign area. The first stage scans the image with sliding windows. Afterwards, the second stage classifies these proposals into corresponding categories as the Mask R-CNN is trained on the manually annotated input images and generates masks on the object [23].

The first stage involves two convolutional neural networks, namely the feature pyramid network (FPN) for image representation, and the region proposal network (RPN) to scan FPN. The FPN was built on the feature extractor, which is a standard convolutional neural network. The backbone network can be illustrated in a pyramid shape, which starts with the input image at the bottom [24]. The early layer is converted from the input image and extracts low-level features, for example, blobs and corners. Furthermore, the top feature map layer is converted layer by layer and can detect higher level features like signs and persons. In the Mask R-CNN paper, the backbone network was extended to FPN by adding a second pyramid to represent the objects at multiple scales, allowing features in each layer to connect layers in higher and lower positions. For the generation of object-containing-area proposals, RPN scans over the feature map layers in RPN from top to bottom. It scans as sliding windows on the boxes, which are called anchors. There are about 200,000 anchors of various sizes and aspect rations, and they overlap each other to have as much coverage of the image as possible. RPN locates the bounding box, assigns the object with the image partition by anchors, and generates the anchor class (foreground or background) and the bounding box refinement as outputs. With the RPN prediction outputs, the anchor with the highest foreground score was selected using non-max suppression and the refinement guaranteeing the anchor to have the best fitness to the object. Eventually, the first stage generates the final proposals, which are also called the regions of interest (ROIs) [23].

At the second stage, another convolutional neural network (mcnn in Figure 1) generates the object class for each ROI as well as the bounding box and mask to indicate each object. However, the size of bounding boxes is not standardized due to their generation processes in both stages. To obtain a better performance of ROI classification, this network firstly introduces an ROI pooling technique named ROIAlign to resize the ROI boxes with a bilinear interpolation. Additionally, a further applied mask branch distinguishes the Mask R-CNN method from its predecessor, the Faster R-CNN. Initially, ground-truth masks are down-sampled to compute the loss, and afterwards, the predicted masks are upscaled to the ROI bounding box. Ultimately, this branch generates a mask on the positive regions selected from the ROI classification [23]. An example output is illustrated in Figure 1, where the visible part of the stop sign above the flood is masked and splashed after stage two in this section.

### 2.2. Traffic Sign Segmentation

To measure flood water depth, it is essential to correctly detect and segment the object, as well as the feasibility to interpret the detection results into the ultimate water level data. With one of the effective frameworks in the instance segmentation field, the Mask R-CNN, the first goal can be reached. Such a deep learning method can recognize and detect the object of its type and distinguish one from another even overlapped by each other. In other words, the above-water part of the traffic signs can be segmented in the average flood scenario. We make the following two assumptions:

1. Signs stand still in the flood; and
2. The stop signs in input images have a standard size.

Why do we assume that the images we use only contain signs that stand still? In this study, we focus on the basic feasibility of the method and its possibility to produce flood depth data, which means extreme cases of flood events occurring in urban areas are not considered. Here, extreme cases can refer to floods during which the water level exceeds the total height of stop traffic signs. This can result in images where the stop signs are leaning too much as if they were bent or captured obliquely by the camera. These extreme scenarios in the application phase could be avoided by asking social media users or photographers to follow certain photo shooting guidelines for citizen-uploaded images and excluding the images where stop signs have large displacement from before the flood. Photos are usually used by news media to indicate the flood depth with the intuitive illustration of traffic signs being submerged. Therefore, news photos are mostly not in the extreme flood scenario mentioned above and they are the ideal input data to validate our method.

**Figure 2:**
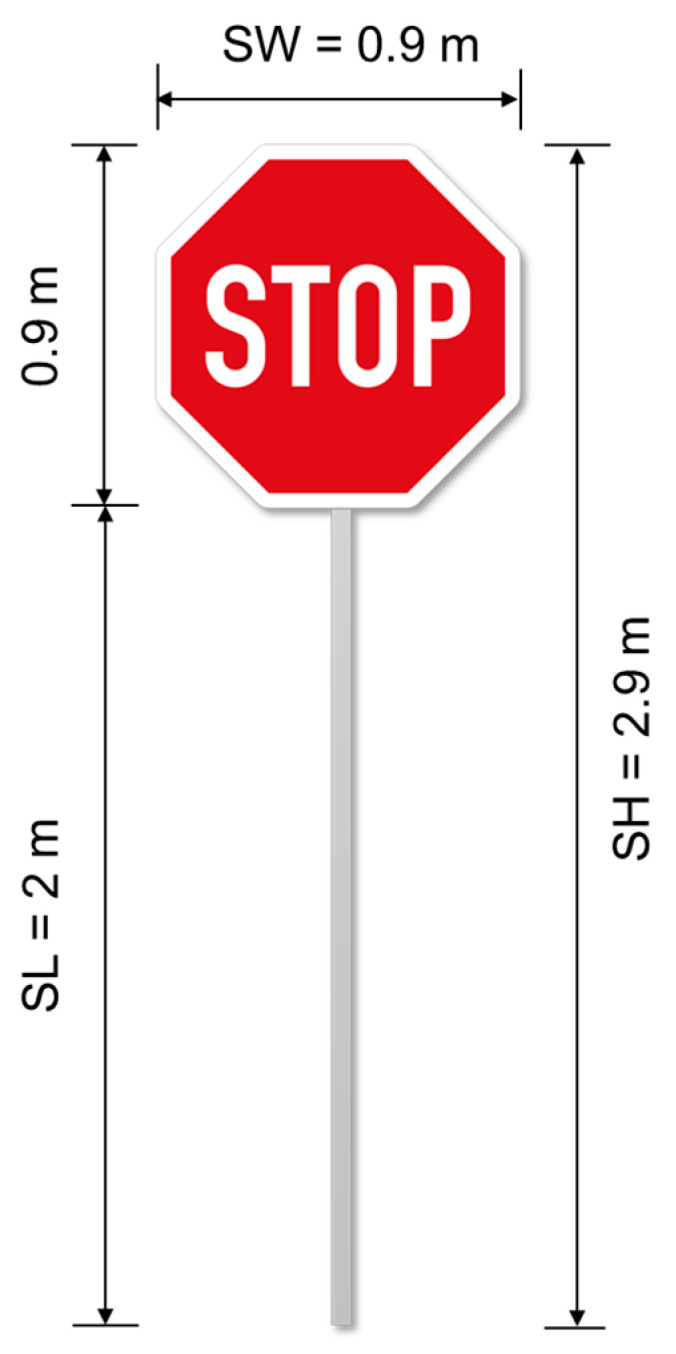
Standard-sized stop signs in this study.

The second assumption is made because the interpretation of segmented traffic sign images requires knowledge of their real size. Usually, one type of traffic sign has a universal size within a certain administrative region [25]. For example, in Germany, stop signs should have a standard size of 900 mm × 900 mm, according to the Germen road regulation Section 1, chapter 41 (Teil 3–Vorschriftzeichen nach Anlage 2 (zu § 41 Absatz 1 StVO)). This standard-sized stop sign is displayed in Figure 2. Moreover, there are industry standards in Germany for setting up devices for standard traffic signs, including their foundations, published by the Güteschutzgemeinschaft Verkehrszeichen und Verkehrseinrichtungen e.V. Therefore, for this academic investigation, the length of the pole part is assumed to be 2.0 m to test our method. In this study, the stop sign is used as an example group of the traffic signs to develop and validate the flood depth monitoring method FloodMask with certain knowledge of local traffic signs’ size. The same method framework could be easily transferred to other traffic signs in future studies.

**Figure 3:**
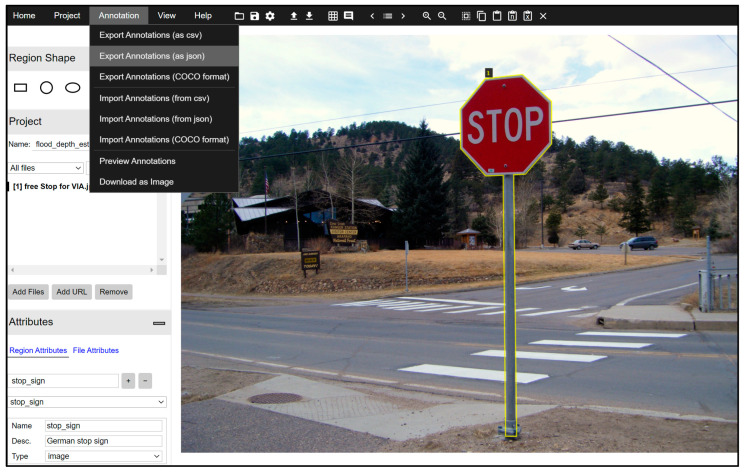
Annotation of stop sign objects on VIA. Detailed link of this image is provided in Supplementary Materials.

Due to constraints from the assumptions and existing resources, only a limited number of images containing stop signs with and without flood were collected from internet as input data. In this study, 80 stop sign images without flood were annotated as the input images for Mask R-CNN training, half of which were used for validation. As shown in Figure 3, the annotation was operated manually utilizing the VGG Image Annotator developed by Visual Geometry Group [23], which is the same as in the Mask R-CNN paper. Once the training process was completed, the experiment was carried out using 16 images with flooded stop signs, where the stop signs were in different conditions. Furthermore, the Mask R-CNN in this method adopted the ResNet101 and Feature Pyramid Network backbone [23]. The source code was implemented using Python 3 and the open-source machine learning platforms TensorFlow and Keras.

### 2.3. Flood Depth Data Extraction

The masked images were used as input in this step to interpret flood water levels with computer vision and basic data processing functions. Generally, the flood depth data retrieval can be concluded in two parts.

The first part deals with obtaining the width and height of the masked sign on the pixel level, as in $W_{p}$ and $H_{p}$. For this purpose, two image processing methods A and B, introduced in Section 2.3.1, were used. The results were simultaneously generated and collected for further analysis. A manual retrieval process was adopted, as presented in Section 2.3.2, for later comparison with the image processing result.

**Figure 4:**
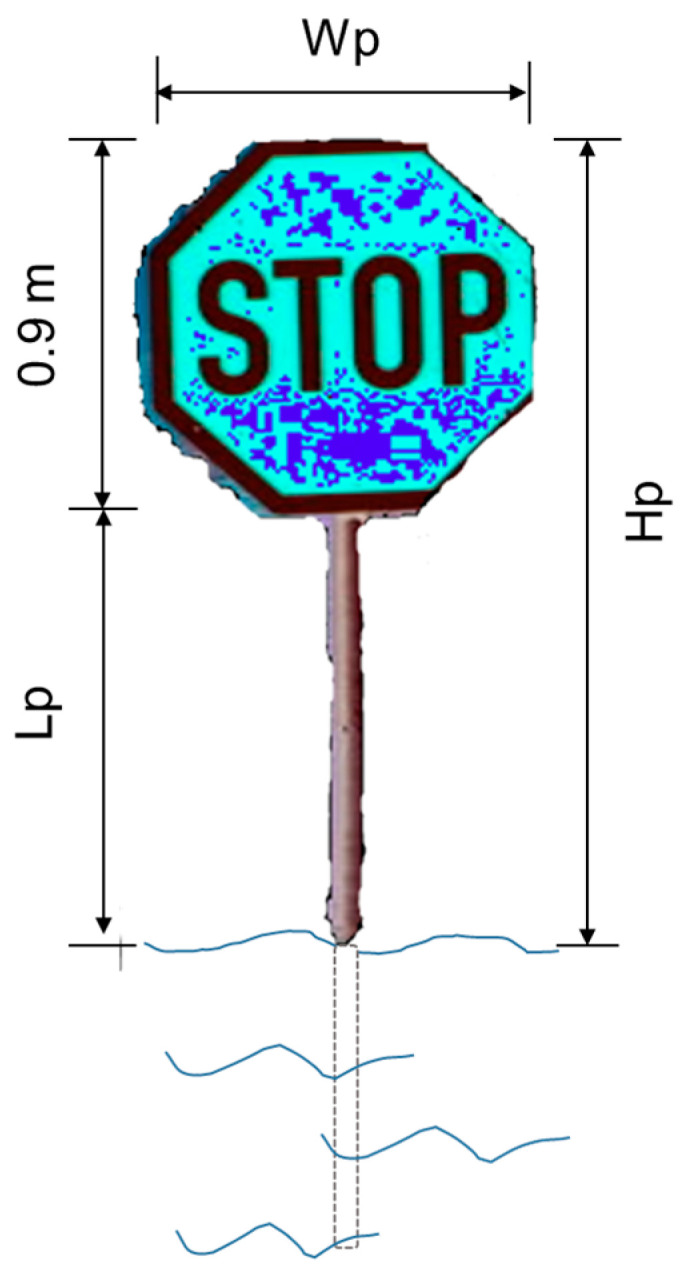
Parameters extracted from the first part of water level retrieval using masked stop sign images in flood. Wp, sign width in pixel; Hp, unflooded sign height in pixel; Lp, unflooded pole length in pixel.

The second part of water level retrieval was a final calculation with all the parameters from the first part to acquire the flood level in a metric unit. This part was applied in both image processing methods (A and B) and the manual retrieval process. For each masked image, the calculation process and relation between the parameters are illustrated in Figure 4. First, $W_{p}$, the width of the masked sign was used to calculate the pixel ratio (r) (Equation (1)), which is defined as the ratio of the same distance in each image to the actual distance in a metric unit, using the width of the stop sign (SW in Figure 2). Furthermore, the pixel ratio can bridge between the pixel height ($H_{p}$) and the actual height ($H_{a}$) of the unflooded sign part, using Equation (2), as the pixel ratio also equal the actual height of the unflooded sign divided by the pixel height. Here, $L_{p}$ and $L_{a}$ were used when the sign pole was available for manual retrieval. In the end, the flood depth in meters was calculated with Equation (3):

(1) $$\frac{W_{p}}{\mathrm{SW}}=r$$

(2) $$H_{a}=r\times H_{p}{\;\mathrm{or}\;L}_{a}=r\times L_{p}$$

(3) $$F=\mathrm{SH}-H_{a}\;\mathrm{or}\;F=\mathrm{SL}-H_{a}\prime$$

where r is the pixel ratio;$\;W_{p}$ is the width of the sign on the pixel level; SW is the standard sign width of the sign panel, 0.9 m; r is the ratio of the distance on the pixel in an image to the actual distance in metric units;$\;H_{p}$ is the height of the unflooded sign part, including the panel on the pixel level;$\;L_{p}$ is the length of unflooded pole on the pixel level;$\;H_{a}$ is the actual height of the unflooded sign part, m;$\;L_{a}$ is the actual height of the unflooded pole part, m; F is the flood depth in metric units, m; SH is the standard height of the whole sign, 2.9 m; and SL is the standard length/*45 of the pole, 2 m.

In the following two subsections, we describe the methodology of image processing and manual extraction in more detail.

#### 2.3.1. Flood Depth Extraction Using OpenCV

**Figure 5:**
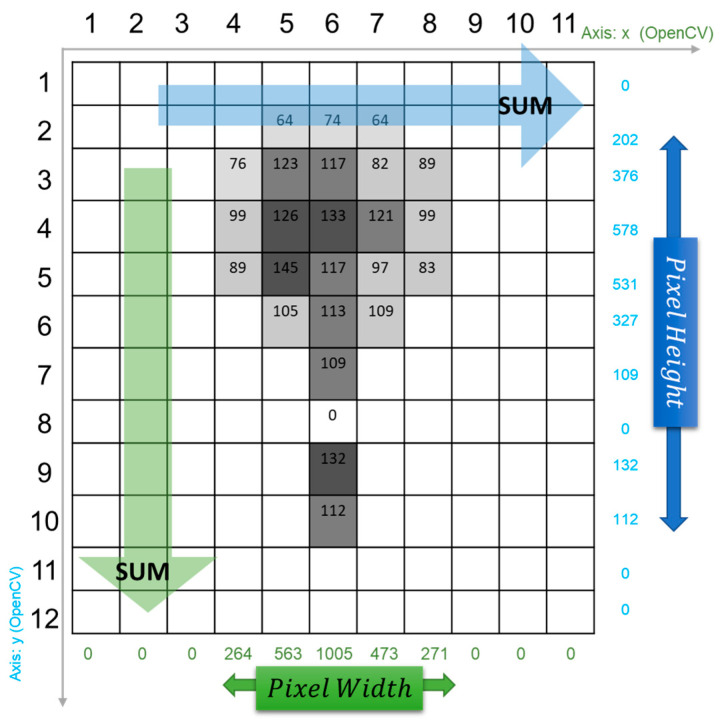
Brief illustration of the masked and flooded stop sign measurement calculation process on the pixel level using Python with a 11 × 12 array.

In the field study of computer vision, the OpenCV (Open Source Computer Vision Library) library has long been established to carry out image processing [26]. Recent development of the OpenCV library has facilitated more advanced real-time application of computer vision [19,27]. In our study, the OpenCV and NumPy libraries were proposed to process the masked images. The utilization of the NumPy library was based on the convenience of image data calculation as an array. The array form is due to the nature of the image coordinate system, which defines each pixel with a certain location in an image. Each pixel may contain several channels to store information. In the early stage, input images that included RGB color information were converted into grayscale. Therefore, the image pixel distribution was presented in a two-dimension Cartesian coordinate system and for each pixel, there was only one grayscale value, making the materials less complicated to analyze and the target feature unchanged (see Figure 5). All the grayscale values were stored in the coordinate array in the shape of the corresponding image size.

After obtaining a grayscale image, image pre-processing was carried out to acquire a better image quality for later image manipulation. In this study, Gaussian blur (also known as Gaussian smoothing) was applied to enhance the image structure, which is a widely used procedure in computer vision algorithms. Since the grayscale masked images could contain noise, random variation in the brightness, or hue among pixels, the grayscale values for pixels showed a high standard deviation. Therefore, a hazier image with a normal distribution of grayscale values provided less random values for later calculation. The Canny edge detector was also applied to detect the edges in images. Such pre-processing procedures were adopted to improve the relative coarse masking and produce output data as precisely as possible. This is especially relevant because the background and flood water surface of stop signs in the image may affect the mask results, incorrectly producing a masked object area with a longer pole, which is a reflection from the water surface.

After the pre-processing, two uncomplicated representation methods were operated to calculate the height and width parameter in the pixel level, which is introduced in Figure 4. As illustrated by Figure 5, the green and blue arrow indicating the grayscale values were added to the column and row in the first place, respectively. Then, we introduced two methods, namely, method A and method B, to deal with the sum values.

(1) Method A: numbers of the non-zero sum values of the column and row, respectively
(2) Method B: W_p_ and H_p_ were counted as one plus the maximum difference of indices of non-zero sum values in the column and row, respectively.

In Figure 5, the non-zero sum values added to the row total nine in the blue color, meaning the pixel height equaled 8 pixels due to one 0 value pixel in the middle. Method B considered input cases in which multiple objects were masked in the image and full and correct detection of the target failed. For example, the smallest index of the non-zero sum value added to the row was the second, and the maximum index was the tenth. Thus, the pixel height should be 10 minus 2 plus 1, which equals 9 instead of 8 from method A. In the illustrative case of Figure 5, these two methods lead to the same results. Yet, the existence of the missed masked or break zones result in method A producing a shorter pixel height or width compared to method B, which eventually resulted in more error in the later flood depth calculation.

Once the pixel height and pixel width were acquired, flood depth data were then computed using Equations (1)–(3) in Section 2.3. Finally, the automated flood depth retrieval with Mask R-CNN and image processing was completed.

#### 2.3.2. Flood Depth Reference Data by Manual Calculation

**Figure 6:**
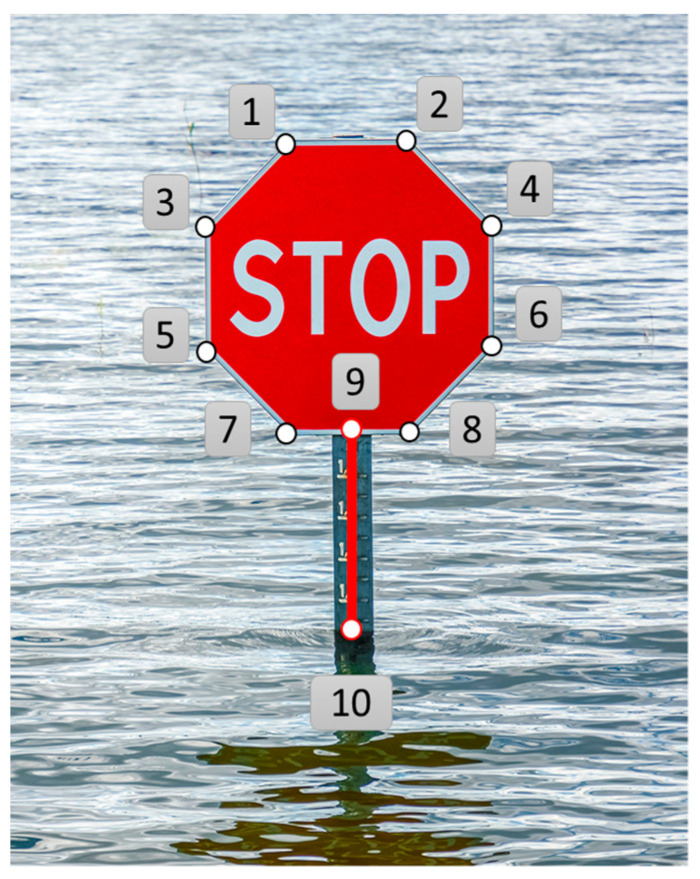
Illustration of the manual retrieval method. The points (1–10) indicate the critical edge for the sign shape and are used for dimension calculation.

As a validation step to output error with image processing data, manual retrieval by human visual inspection was carried out, using unmasked stop sign images with flood. Human visual inspection started with pinning points on the essential location that shaped the object. In Figure 6, the points are located with the same order and their coordinates were displayed directly in image editing software like “Paint”, when putting the mouse cursor on the points and then recorded. Point 9 and 10 located the top and bottom ends of the unflooded pole part. In particular, points 7, 8, 9, and 10 were not visible in some images, as shown in Figure 6, due to the various flood scenarios captured. Hence, images with over-pole flood required a slightly different calculation process for the pixel width and height.

After obtaining the coordinates, the parameters of the masked area mentioned in Equations (1)–(3) were calculated to obtain the flood depth results. At first, $W_{p}$, the width of the panel, was calculated with the dropped points of 3, 4, 5, and 6 with Equation (4), which is the average of the x coordinate difference between 3 and 4, as well as 5 and 6. If the points 5 and 6 are submerged and not shown in the panel, then point 5 and 6 were placed by dropping the points at the intersection location of the flood surface and panel. Then, $L_{p}$, the height of the unflooded pole on the pixel level, was calculated as the Euclidean distance between point 9 and 10 using Equation (5). When the pole was entirely inundated, $H_{p}$, the height of the unflooded sign, was calculated with Equation (6) to determine the ratio of pixel and metric system:

(4) $$W_{p}=\frac{\left(x_{4}-x_{3}\right)+(x_{6}-x_{5})}{2}$$

(5) $$L_{p}=\sqrt{{\left(x_{9}-x_{10}\right)}^{2}+{\left(y_{9}-y_{10}\right)}^{2}}$$

(6) $$H_{p}=\max\left(\left(y_{10}-\left(y_{1}+y_{2}\right)/2\right),\left(\left(y_{7}+y_{8}\right)/2-\left(y_{1}+y_{2}\right)/2\right),\left(\left(y_{5}+y_{6}\right)/2-\left(y_{1}+y_{2}\right)/2\right)\right)$$

where $x_{n}$ is the x coordinate of point n as in Figure 6 and $y_{n}$ is the y coordinate of point n as in Figure 6.

Finally, the flood depth for each image was calculated using Equations (1)–(3) with manually measured parameters on the pixel level. Thus, the flood depth data were retrieved for later analysis. For evaluation of FloodMask, manually retrieved data were used to compare and analyze the error.

One noticeable limitation of projection distortion arises from the parameter calculation methods (in Equations (1)–(6)): the signs might distort when the input images in flood are not in orthographic projection in the camera, resulting in the calculated flood depth data being larger or smaller than the ground truth for each case. Perspective transform of the traffic sign could be achieved using another convolutional neural network with boundary estimation of the detected object [28]. This method requires specific shape labels in the training dataset, which is not available in the existing public traffic signs training datasets, such as the German Traffic Sign Recognition Benchmark [29]. Preparing such shape labels involves a considerable amount of manual work and might introduce potential subjective bias. Moreover, a reasonable assessment of the distortion effect requires on-site flood depth measurements. However, the timely flood depth measurements are rare and unobtainable, especially for photographer-taken and social media images. Therefore, the impact of distortion needs specific investigations and is a standalone research topic that is beyond the scope of this study. In this work, we focused on improving the efficiency to derive flood depth data in an automated manner in comparison to traditional manual measurements. The reference flood depth was calculated from visual perception data on the original images regardless of distortion projection. More importantly, images with extreme distortion were not adopted when collected manually online. Future studies could set up an experimental site to record the actual flood depth and collect flood images for accuracy investigation.

## 3. Results and Discussion

In Section 3.1, Mask R-CNN-generated results in the corresponding masked images where the segmented objects are highlighted. Additionally, preliminary steps generated an annotation file and the trained convolutional neural network. Next, flood depth data detected by both image processing and manual retrieval are presented in Section 3.2.

In reviewing the literature, no such quantitative method was proposed for the collection of flood depth data. Previous studies using a similar machine learning algorithm only estimated the flood level based on the flooded objects, including humans walking through the flood and house, which is affected by various uncertainty sources [22]. In our study, an initial objective was to investigate the feasibility of this methodology with convincing data interpretation logic. Moreover, this study set out to assess its application scenarios. The third question in this study sought to determine its accuracy under the suitable application scenarios. In the following sections, the results are discussed in two parts, including the Mask R-CNN performance and flood depth retrieval outputs.

### 3.1. Instance Segmentation

**Figure 7:**
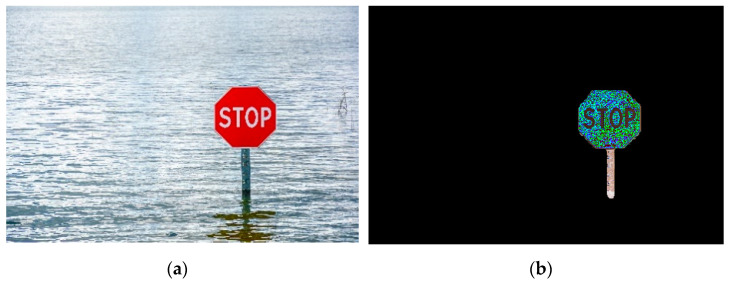
Illustration of the regular scenario: (**a**) the input image; (**b**) the output image.

**Figure 8:**
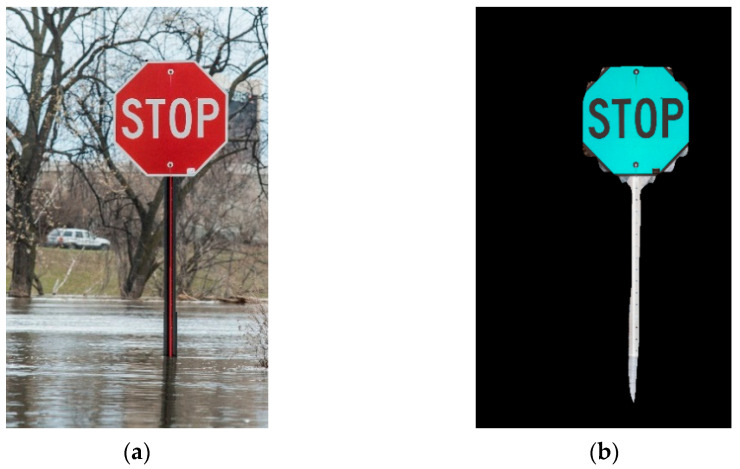
Illustration of the mirror reflection scenario: (**a**) the input image; (**b**) the output image with an extended masked object.

**Figure 9:**
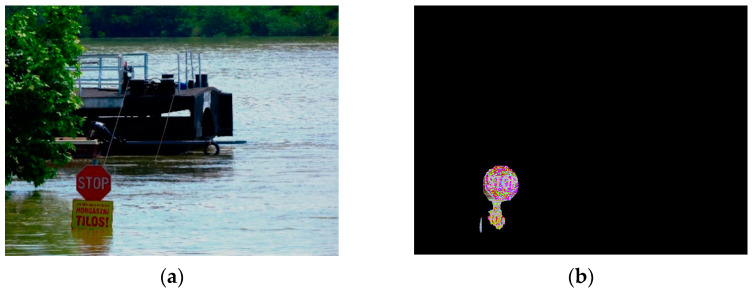
Illustration of the multi-object scenario: (**a**) input image; (**b**) output image.

First, to establish the training materials, 80 stop sign images without flood were annotated and a JSON file containing the annotation and category information was produced. Then, in the main Python script of execution, the JSON file was used to train the convolutional network. Second, the network learned from the training images and then was established after training and validation steps. Third, the network segmented and masked the input images with flood and finally, produced an PNG image (see Figure 7b, Figure 8b, and Figure 9b, respectively) for each input JPG image (see Figure 7a, Figure 8a, and Figure 9a, respectively).

Figure 7, Figure 8 and Figure 9 illustrate selected examples in various situations. Generally, three scenarios were classified to describe the interaction between the stop sign and the flood shown in the input images for FloodMask, including the regular scenario, multi-object scenario, and mirror reflection scenario. Figure 7 shows the regular scenario where the sign is partly flooded, and the input image holds a simple background. The output masked target in Figure 7b is relatively clean and clear and without obvious visual precepted problems, especially the types of problems in the following scenarios.

Problematic scenarios were due to various factors, such as the intricacy of the background, object condition, and image quality. One problematic mirror reflection scenario example is demonstrated in Figure 8, where the mirror reflection of the sign leads to an extended pole mask in the output. Human visual inspection indicated that there is a difference between the above-water object and its noisy reflection. Yet, the Mask R-CNN considered the part of the reflection of the pole as an entity. The detected flood depth was usually underestimated according to how long the mirror reflection was masked. In Figure 7, the flood depth from FloodMask for both method A and B in image processing is shown as 0.13 m, which is 0.46 m less compared to the manual retrieval result of 0.59 m.

Results from the multi-object scenarios are shown in Figure 9. What stands out in Figure 9a is that there is an extra sign beneath the stop panel. It can be seen from Figure 7b that the stop panel was successfully detected, and the rectangular text was also partly masked. The margin of the masked rectangular text sign still outlined the flood position on the sign, but the width was expected to be affected. The FloodMask determined a flood depth of 1.29 and 1.34 m for methods A and B, respectively, and the error decreased to 0.04 and 0.08 m. Another situation causing flawed results could be an extra sign masked next to the target object STOP sign. As a result, the Mask R-CNN generated the mask on the wrong category of the object. In this scenario, the target was masked but with an excessive width and unacceptable results. For this case, a multiple sign filter was applied in water level data retrieval, which recognized and discarded the masked images in this case by judging whether the non-zero values on the *x*-axis direction have a break area, indicating that there might be several objects masked.

With respect to the first and third research objectives, it was found that the user-trained Mask R-CNN successfully detected the object in the image. In the Mask R-CNN paper, the average precision was relatively low for certain categories like trucks and trains, indicating a domain shift presence when training data is lacking [23]. Consistent with the Mask R-CNN literature, this research found that objects from other categories are mistaken as the target object and masked as output. Another possible explanation for this negative result may be the lack of adequate training samples with high image quality and different image sizes.

Nevertheless, with the small amount of 77 input images, caution must be applied, as the uncertainty analysis according to the current results might not be comprehensive. Therefore, to develop a more efficacious method of instance segmentation, additional studies will be needed, which could utilize more abundant training samples or further state-of-the-art machine learning algorithms.

### 3.2. Flood Depth Data Retrieval

We turn our attention now to the water level retrieval results using the masked images from the last section. The manual retrieval data is demonstrated in three scenarios, where the first two groups represent the ideal situation, and the other two groups indicate the problematic situations as mentioned before. Then, the results continue with the data from the image processing step. In the end, the comparison and analysis results are presented to evaluate FloodMask.

There are nine images in the regular scenario presenting a difference between the results errors of method A and B in FloodMask (Table 1). This is quite revealing in several ways. Even though method A generated marginally less error than method B in a few images, method B still outperforms method A with an average error of 0.17 m rather than 0.36 m for method A. The most obvious finding to emerge from the analysis is that it is feasible for the automated method to detect and interpret flood depth data with satisfying precision, which thus answers the first research objective. A 0.17 m error could have originated in the image processing procedure, where images with poor quality may lead to coarse edges in the masking step. While this limitation should be considered when interpreting the results, the error is still significantly better than most of the existing water level data. However, such an underperformance could be eradicated in method B using array indices to measure the masked area. Hereafter, the following analysis is presented with the method B results.

**Figure 10:**
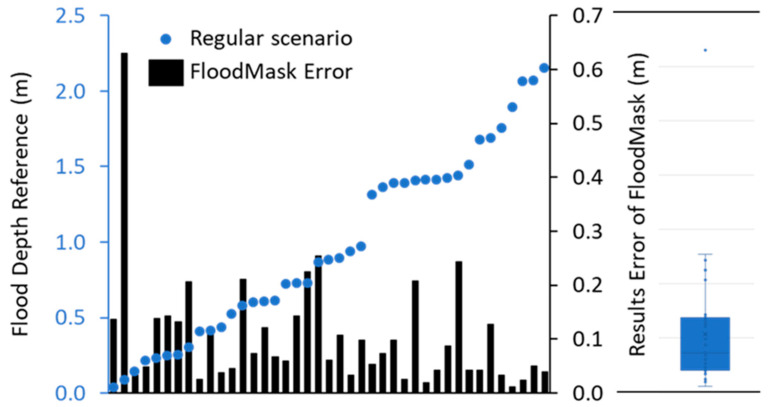
Visual perception flood depth reference data and the error of the FloodMask results with a boxplot in the regular scenario.

**Figure 11:**
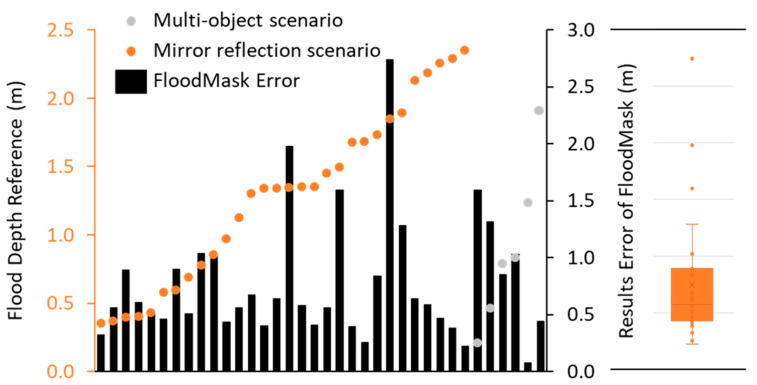
Visual perception flood depth reference data and the error of the FloodMask results with a boxplot in the multi-object scenario.

Figure 10 and Figure 11 present the flood depth output of FloodMask in errors and the visual perception reference data for all 77 images. There are 41 images in the regular scenario with an average error of 0.11 m, including 7 cases where the sign pole above the flood was not completely segmented, which still largely outperformed those in the problematic scenarios. Figure 10 illustrates that there is significant bias from the reference in the mirror reflection scenario with an average error of 0.75, which varies from 0.25 m to as high as 2.74 m. Most of the results in this case underestimated the flood depth and where the falsely extended pole area on the flood water surface is masked by FloodMask. These results match with the estimation with the intuitive inspection, as humans can easily distinguish the reflection from reality based on the image information and the knowledge of symmetry while Mask R-CNN cannot. Moreover, the multi-object scenario images also led to results with dramatic error of up to 1.60 m, as shown in Figure 11. The multi-object scenario reveals that the segmentation results can largely affect the final precision, but images with this scenario could be excluded with the application of a multi-object filter in FloodMask codes.

Figure 10 and Figure 11 contain boxplots of the two scenarios’ errors of the flood depth results from FloodMask, derived from the visual perception reference data. The result of the regular scenario has the most compact box, and its whisker is shorter on the smaller error level, indicating a positively skewed distribution with only one outlier due to a mask failure that the sign pole is not fully segmented. In the mirror reflection scenario, the two distancing whisker ends and the three outliers indicate a large dispersion of the error data, as the reflection can be extremely long on a smooth water surface. For the multi-object scenario, the image sample size is too small (only 6) to make any statistical results and they can be excluded.

### 3.3. Outlook for Practical Use

The mirror reflection problem may lead to large and dispersive error in application (in Section 3.2). However, there is abundant room for further progress to remove the reflection by taking images with a polarization lens to filter the light in the image at the reflection area or set up image collecting rules for social media image resources. For some cases, the pole color may be easily distinguished from the reflection and thus can by removed with further image prepossessing steps. Additionally, for multi-object images, a filter could be applied in image processing procedure to excluded cases when multiple masks appear, reducing the influence of such errors on the average results as presented in Section 3.2. Moreover, caution should be exercised for the distortion projection in the extreme condition (see Section 2.3.2) in which error may be considerable large. In the application of FloodMask, the ideal input flood image should be collected from a rather orthogonal perspective by guiding the users when taking photos and using an onboard lens on passenger vehicles.

For practical use, the proposed flood depth measuring frame could be easily transferred to any traffic signs with a uniform size, which is set by the local government or industry for a country or a state. The limiting factor, however, is the availability of photos with flood and traffic signs together. Though there are many such images on social media or photo databases online, most of them are protected by copyright, complicating their usage. This could be overcome by public service departments establishing camera-based monitoring systems, such as traffic CCTV (closed-circuit television camera) networks, in flood-sensitive regions. Individual parameterization of post-processing could be set up for each monitoring location by the user, which would improve the accuracy of the depth estimates due to the relatively static backgrounds. Besides, business entities, like vehicle companies and map service companies, could collect on-board vehicle camera information with consent and provide a flood warning service. Moreover, compared to other traditional measuring sensors like remote sensing, gauge stations, and onsite sensors, this image-based method has serval advantages: it works faster, records the evidence (i.e., image) for double checking, and uses traffic CCTV resources without the cost of installation of extra monitoring devices.

## 4. Conclusions

This study set out to provide an automated methodology to train a convolutional neural network to be able to segment stop signs and retrieve flood depth data from flood images that include such signs. The main goal was to examine the feasibility and performance of this methodology, investigating the extent to which it can be applied to different scenarios of input images. Based on the results of this study, this method can detect the target objects and interpret the flood depth with a satisfying precision of error of 0.11 m for images without mirror reflection and multi-object scenarios. 

One of the strengths of this study is that it represents an investigation of the potential to apply machine learning algorithms and computer vision in the field of hydrological data acquisition. It offers an exploration of such a framework and yields an affirmatory answer. Another strength of FloodMask lies in its potential to automate an expansion of the flood depth data source. Here, the empirical findings provided a new understanding of the potential resources of water level data for hydrological modeling. It expanded the water level data resources to social media photos, surveillance footage, and even on-board vehicle cameras other than those generally utilized data sources, including remote sensing data, gauge station records, and ground sensors. Moreover, this method established an automated way, which could save labor costs to some degree and increase the efficiency of the data processing procedure.

The generalizability of these results is subject to certain limitations. For instance, the sample was regionally representative of the stop sign but gave no guarantee that all other categories of traffic signs in practice have a standard size in reality. Furthermore, with a small training sample size, caution must be applied, as the instance segmentation results may not be transferable to other training samples. However, an increasing number of datasets will be available for application. For example, an open-access traffic sign dataset of German road signs was prepared with more than 50,000 images and in 43 classes, and on which CNNs produced high accuracies for classification [29]. Additionally, mirror reflection provides an intriguing problem that could be explored in further research, as more information on this problematic scenario would help us to establish a greater degree of accuracy on this matter. Above all, regional flood depth data analysis and correction is necessary for practical use.

## Figures and Tables

**Table 1:** Flood depth error results of the comparison of method A and B in FloodMask in the regular scenario (meter).

Image	1	2	3	4	5	6	7	8	9	Avg.
Method A	0.72	1.20	0.41	0.24	0.08	0.14	0.08	0.12	0.24	0.36
Method B	0.11	0.63	0.12	0.14	0.02	0.08	0.07	0.10	0.25	0.17

## Data Availability

The flooded stop sign images for validating the method were collected from social media or photo databases online, most of them are protected by copyright. Details links of all validation images are provided in Supplementary Materials. In particular, Figure 3 includes the image from website Flickr and licensed by https://creativecommons.org/licenses/by/2.0/, accessed on 1 April 2021. The original image in Figure 8a is from website Flickr with public domain copyright. The original images in Figure 6, Figure 7a and Figure 9a are bought from website Shutterstock with a standard license of https://www.shutterstock.com/license, accessed on 1 April 2021. Detailed links are provided in Supplementary Materials.

## Supplementary Materials

The following are available online at https://www.mdpi.com/article/10.3390/s21165614/s1.
