# Supplementary material for: Automated Flood Depth Estimates from Online Traffic Sign Images: Explorations of a Convolutional Neural Network-Based Method
Source: Sensors (Basel). 2021 Aug 20;21(16):5614. doi: 10.3390/s21165614 (PMC8402382; doi:10.3390/s21165614)
Supplement: Supplementary file 1 [file sensors-21-05614-s001.zip › sensors-1187691-supplementary.pdf]

1. Image for VIA illustration: Figure 3 in manuscript

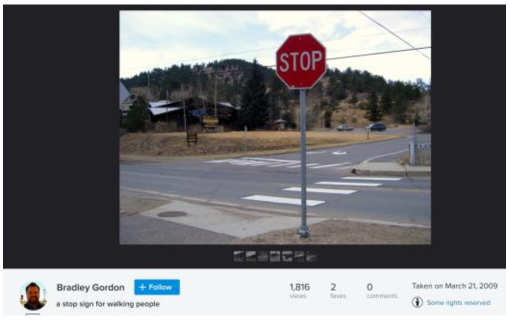

https://www.flickr.com/photos/icanchangethisright/3542372195/

2. Validation Images:

There are 77 images used here in total. The stock_xx images shown in the manuscript are brought and downloaded from the website Shutterstock with a standard license (https://www.shutterstock.com/license)

stock_02: Figure 9a in manuscript

https://www.shutterstock.com/de/image-photo/flooding-on-danube-river-high-water-1416102917

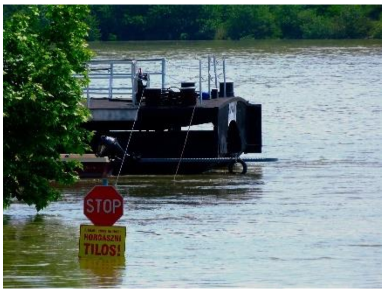

stock_03

https://www.shutterstock.com/de/image-photo/flooded-roadway-226943815

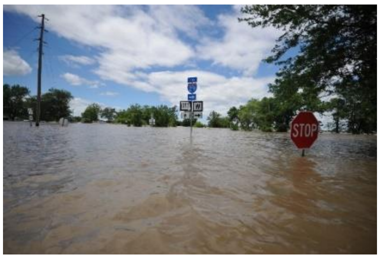

stock_05: Figure 6 and 7a in manuscript

https://www.shutterstock.com/de/image-photo/flooding-road-sign-priority-without-stopping-1216800709

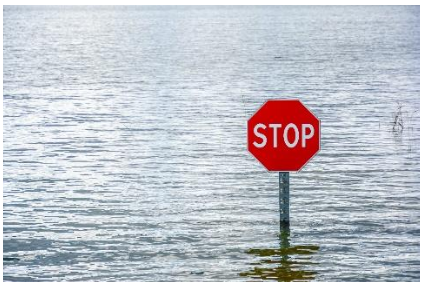

stock_06

https://www.shutterstock.com/de/image-photo/red-stop-sign-lake-that-measures-1905737941

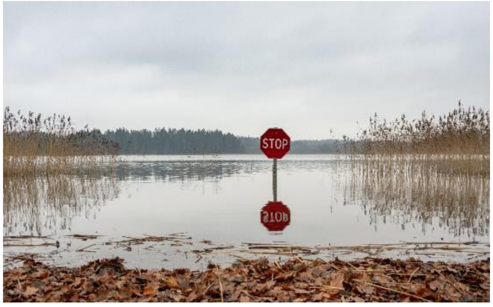

stock_11

https://nara.getarchive.net/media/a-stop-sign-in-the-small-northern-california-town-of-meridian-stands-as-a-sign-161554

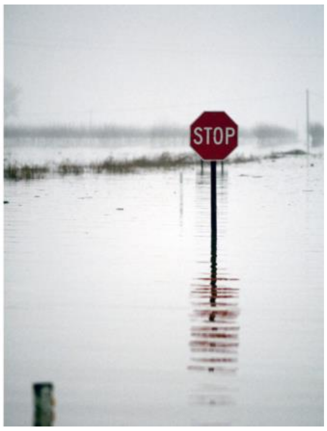

Ⓒ **Copyright info**

No known copyright restrictions

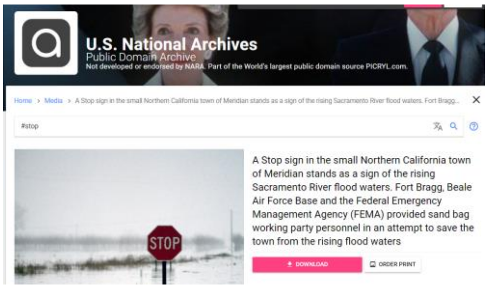

imageA02: Figure 8a in manuscript with public domain

https://www.flickr.com/photos/usdagov/8705067552/

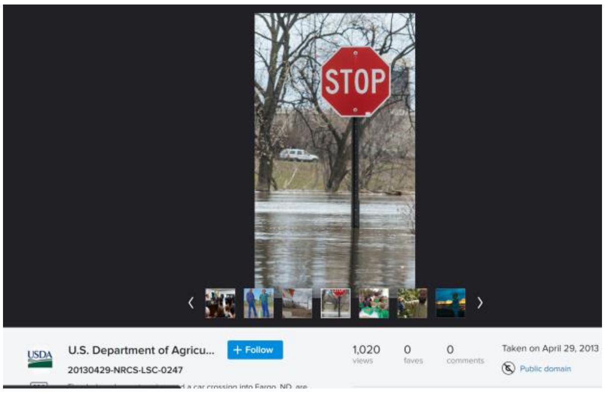

IMAG01

https://media.gettyimages.com/photos/signs-at-a-morring-standing-under-the-waters-of-the-elbe-river-in-30-picture-id982591752?s=612x612

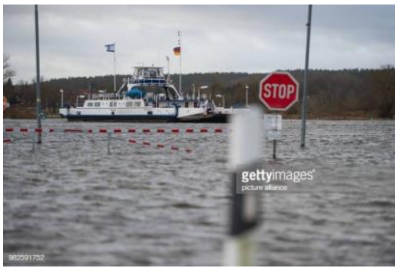

IMAG02

https://www.gettyimages.ie/detail/news-photo/flooded-road-sign-is-seen-on-june-7-2013-in-darchau-afp-news-photo/170154839

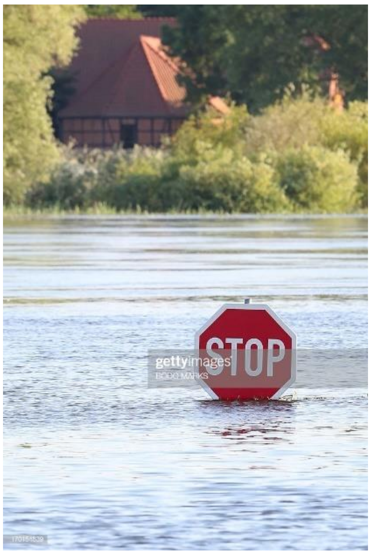

IMAG03

https://st.depositphotos.com/1203257/2741/i/450/depositphotos_27410681-stock-photo-car-under-water.jpg

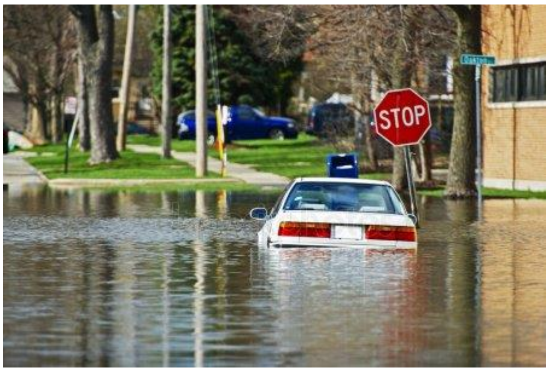

IMAG04

https://thumbs.vienna.at/?url=https://www.vienna.at/2013/06/ABD0257-201306021.jpg&w=1810&h=1358&crop=1

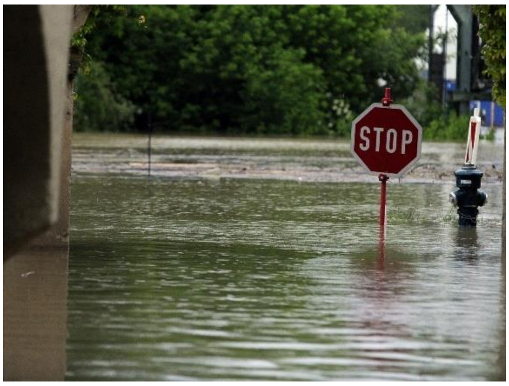

IMAG05

http://www.vulkane.net/blogmobil/wp-content/uploads/2013/06/P1030251.jpg

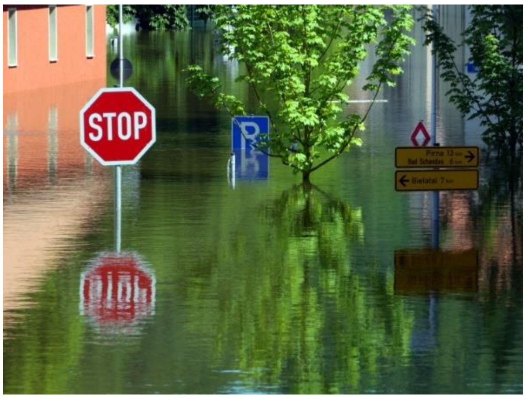

IMAG06

https://img01.lachschon.de/images/153130_RichtigboesesWasser_1-medium.jpg

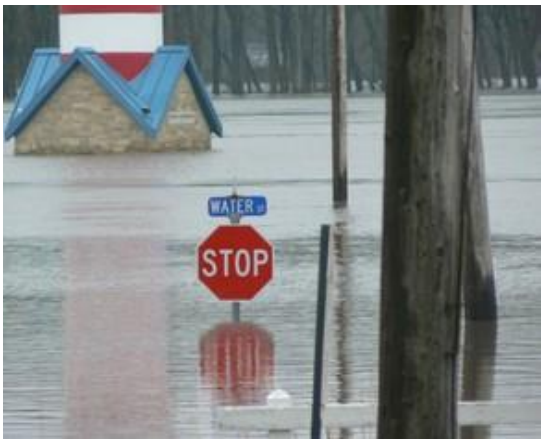

IMAG07

https://www.generali.at/fileadmin/media/presse/bilder/2015/csm_hochwasser_d1c03f43ad.png

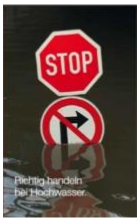

IMAG08

https://www.br.de/br-fernsehen/sendungen/euroblick/bosnien_verkehrschild_hochwasser-100~_v-img__16__9__l_-
1dc0e8f74459dd04c91a0d45af4972b9069f1135.jpg?version=6186d

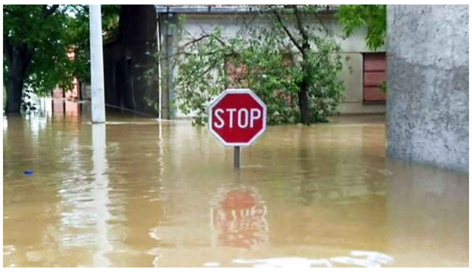

IMAG09

https://media-cdn.sueddeutsche.de/image/sz.1.1477796/1200x675/hochwasser.jpg?v=1522721988

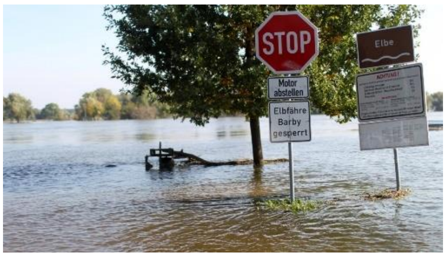

IMAG10

https://www.tagesschau.de/multimedia/bilder/sendungsbild439426~_v-gross4x3.jpg

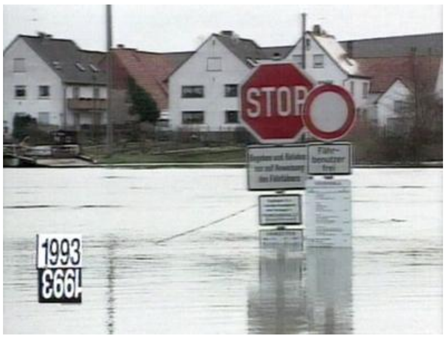

IMAG11

https://www.noz.de/article/teaser/301624/full

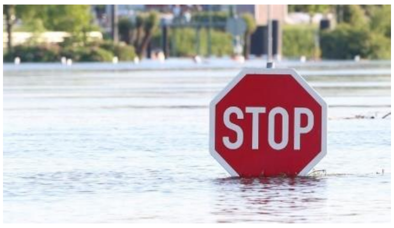

IMAG12

https://media.diepresse.com/images/uploads_620/7/d/8/1415128/954080D6-3311-4819-BC68-71583D8EBD85_v0_h.jpg

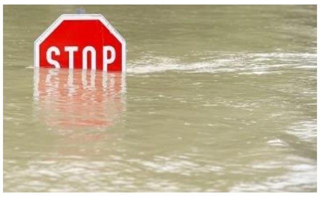

IMAG13

https://www.mz-web.de/image/966788/2x1/940/470/19f79f2677896ad7475e4630f16bb68e/GT/anlegestelle-aken-jpg.jpg

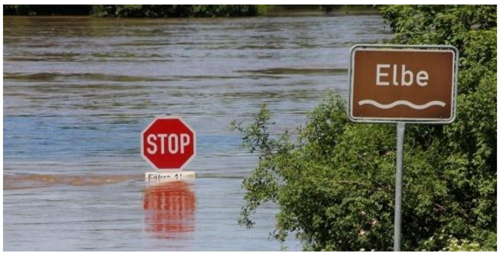

Mimage001

https://whyy.org/articles/shore-town-installs-numerous-flood-sensors-to-improve-storm-resiliency/

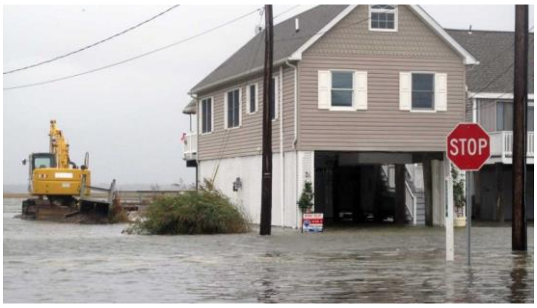

Mimage002

https://www.cbs42.com/wp-content/uploads/sites/81/2020/02/IMG_1819.jpg

https://www.google.com/url?sa=i&url=https%3A%2F%2Fwww.cbs42.com%2Fweather%2Fsevere-weather%2Fphotos-flooding-in-central-alabama%2F&psig=AOvVaw1Ycd59rffNZACzHrekxEFS&ust=1618666907393000&source=images&cd=vfe&ved=2ahUKEwjt9Luh8oLwAhWl67sIHT9VARQQjRx6BAgAEAc

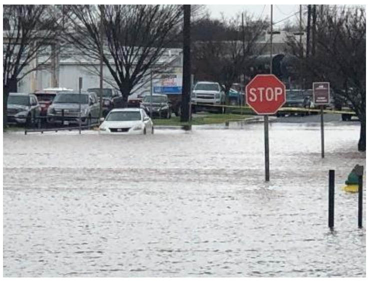

Mimage003

https://www.google.com/url?sa=i&url=https%3A%2F%2Fwww.cbsnews.com%2Fnews%2Fdangerous-weather-eastern-united-states-flood-watches-latest-forecast-2018-07-25%2F&psig=AOvVaw3n0Z98Jz1Wo1YObLXKjN2C&ust=1618666901354000&source=images&cd=vfe&ved=2ahUKEwiqrsue8oLwAhWF_bsIHarNDbMQjhx6BAgAEA4

https://cbsnews2.cbsistatic.com/hub/i/r/2018/07/25/356083ac-e979-441e-b59f-0a1be63e06bc/thumbnail/1280x720/edc16feb480c7be03d061ea3c132f0f3/cbsn-fusion-dangerous-flooding-hits-pennslyvania-after-days-of-rain-thumbnail-1620522-640x360.jpg

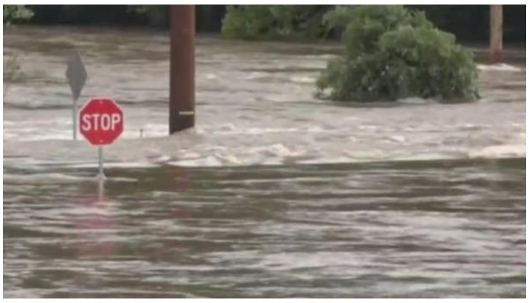

Mimage004

https://twitter.com/nestatepatrol/status/1106220570570567680

https://www.google.com/url?sa=i&url=https%3A%2F%2Ftwitter.com%2Fnestatepatrol%2Fstatus%2F1106220570570567680&psig=AOvVaw1t85hhB9M3AoGJC74lSjYF&ust=1618666899061000&source=images&cd=vfe&ved=2ahUKEwjBur-d8oLwAhV8hv0HHcwqDE8Qjhx6BAgAEA4

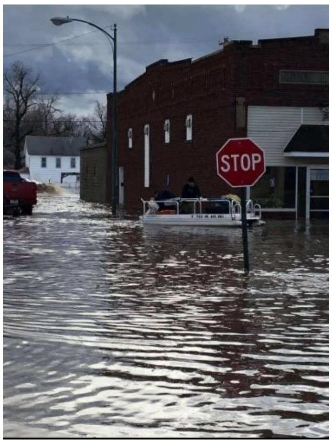

Mimage005

https://www.nbc4i.com/wp-content/uploads/sites/18/2019/06/waldo-flood-rescue.jpg?w=1280

https://www.google.com/imgres?imgurl=https%3A%2F%2Fwww.nbc4i.com%2Fwp-content%2Fuploads%2Fsites%2F18%2F2019%2F06%2Fwaldo-flood-rescue.jpg%3Fw%3D1280&imgrefurl=https%3A%2F%2Fwww.nbc4i.com%2Fhome%2Fsemi-passes-signs-gets-stuck-in-flood%2F&tbnid=ad8NUYBgO3velM&vet=10CJoBEDMorQFqFwoTCICJnffvgvACFQAAAAAdAAAAABAD..i&docid=S00kLfMms88uiM&w=758&h=359&q=stop%20sign%20flood&ved=0CJoBEDMorQFqFwoTCICJnffvgvACFQAAAAAdAAAAABAD

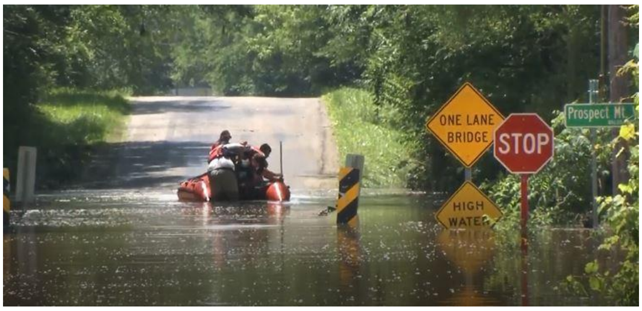

Mimage006

https://www.google.com/url?sa=i&url=https%3A%2F%2Fmwfbic.com%2Fflood-preparedness-safety%2F&psig=AOvVaw1GpCbU6sNHGwEZaxTGTV3N&ust=1618666844311000&source=images&cd=vfe&ved=2ahUKEwjv4rGD8oLwAhUUxrsIHfd6Dy4QjRx6BAgAEAc

https://mwfbic.com/wp-content/uploads/2019/04/Flooding.jpg

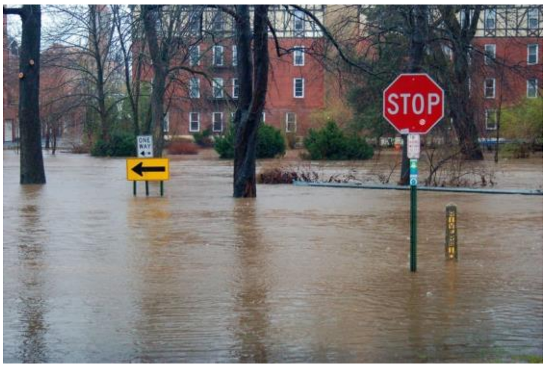

Mimage007

https://www.bemidjipioneer.com/incoming/article3570634.ece/alternates/BASE_LANDSCAPE/43172%2B201003210321-Flood-ThreatMads.jpg

https://www.google.com/url?sa=i&url=https%3A%2F%2Fwww.bemidjipioneer.com%2Fnews%2F240208-red-river-flooding-oslo-island-again-bridge-connecting-climax-buxton-closes&psig=AOvVaw3ZZelvcHN_Wkpe8GInnLEb&ust=1618935324886000&source=images&cd=vfe&ved=2ahUKEwjg1POY2orwAhWI4bsIHb_YBLAQjRx6BAgAEAc

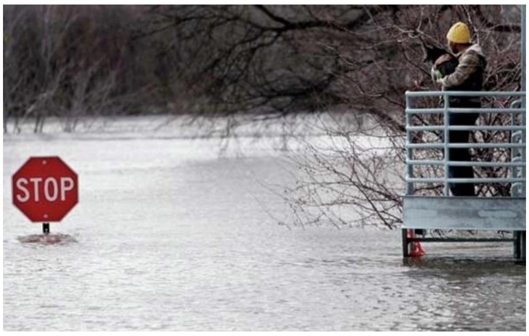

Mimage008

https://www.wlbt.com/resizer/yw5RDXieKa2uUlwUlr1laq3xC1s=/1400x0/arc-anglerfish-arc2-prod-raycom.s3.amazonaws.com/public/2SSHWDS4PRESFEQAVVNRYORNII.jpg

https://www.google.com/url?sa=i&url=https%3A%2F%2Fwww.wlbt.com%2Fstory%2F36534688%2F7000-flooded-cars-are-on-the-roads-or-for-sale-in-mississippi&psig=AOvVaw35zml9KIUhUDODN-cTr8wp&ust=1618666791802000&source=images&cd=vfe&ved=2ahUKEwif6qzq8YLwAhWUhP0HHb1xBb8QjRx6BAgAEAc

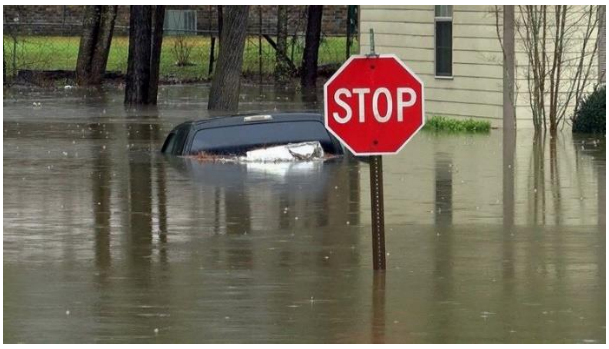

Mimage009

https://media.gettyimages.com/photos/stop-sign-is-seen-at-a-flooded-intersection-on-october-7-2015-in-picture-id491723890

https://www.google.com/url?sa=i&url=https%3A%2F%2Fwww.gettyimages.com%2Fdetail%2Fnews-photo%2Fstop-sign-is-seen-at-a-flooded-intersection-on-october-7-news-photo%2F491723890&psig=AOvVaw2DvPohGCHbVBzCG7glSr2_&ust=1618666737094000&source=images&cd=vfe&ved=2ahUKEwiM2gHQ8YLwAhWnw7sIHSLDBz0QjRx6BAgAEAc

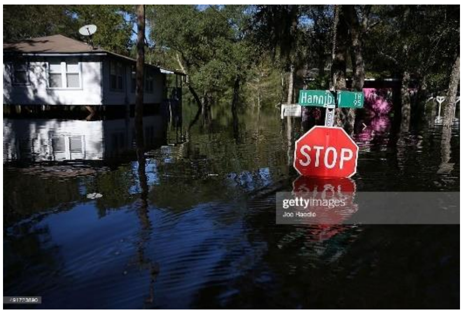

Mimage010

https://dfw.cbslocal.com/wp-content/uploads/sites/15909545/2018/07/flooding-926386668.jpg?w=770

https://www.google.com/url?sa=i&url=https%3A%2F%2Fdfw.cbslocal.com%2F2018%2F10%2F08%2Fflood-sweep-away-west-texas-rv-park-at-least-4-missing%2F&psig=AOvVaw2uk6xP7NrY7Cj1NTnnVphG&ust=1618666733582000&source=images&cd=vfe&ved=2ahUKEwiqt8vO8YLwAhWBxLsIHQO3DiUQjRx6BAgAEAc

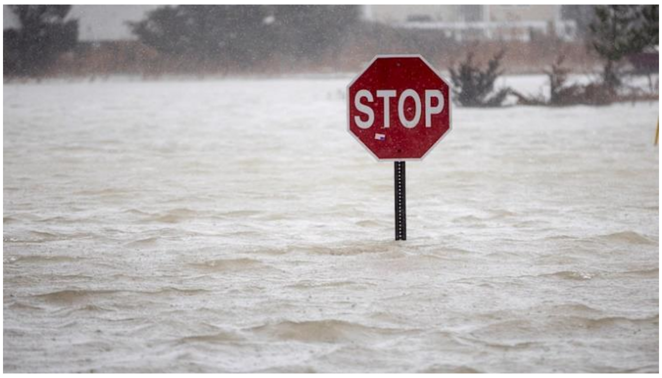

Mimage011

https://www.mccloyconsulting.com/wp-content/uploads/2017/03/stock-photo-stop-sign-in-flooded-river-33550786.jpg

https://www.google.com/url?sa=i&url=https%3A%2F%2Fwww.mccloyconsulting.com%2Fflood-risk-assessment%2Fstock-photo-stop-sign-in-flooded-river-33550786%2F&psig=AOvVaw3aTaJJNkNd2WfaQrHBQ75e&ust=1618666699735000&source=images&cd=vfe&ved=2ahUKEwj1wbm-8YLwAhV5ybsIHecoBGcQjRx6BAgAEAc

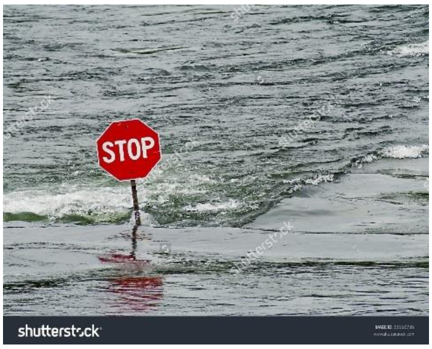

Mimage012

https://www.click2houston.com/resizer/MRZK213N2jUoz_k5E27Ujpje70A=/508x284/smart/filters:format(jpeg):strip_exif(true):strip_icc(true):no_upscale(true):quality(65)/cloudfront-us-east-1.images.arcpublishing.com/gmg/M5JUCYNMNZBVVKV6A2CJ5PLECI.JPG

https://www.google.com/url?sa=i&url=https%3A%2F%2Fwww.click2houston.com%2Fweather%2F2020%2F05%2F11%2Fwhy-we-have-to-watch-for-flooding-this-weekend%2F&psig=AOvVaw2pNbrt0TXob4U8WxhlzLdU&ust=1618666695884000&source=images&cd=vfe&ved=2ahUKEwiYvc688YLwAhW16rsIHTuADagQjRx6BAgAEAc

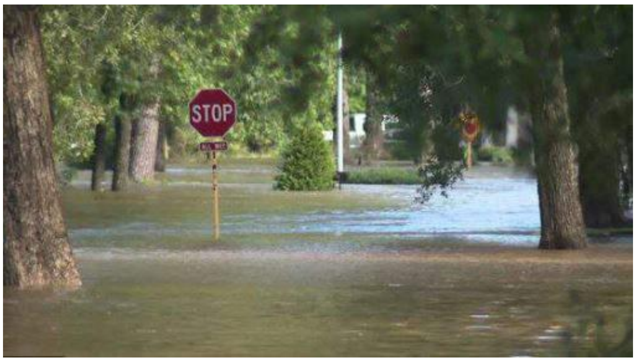

Mimage013

https://www.google.com/url?sa=i&url=http%3A%2F%2Fmommasaid.net%2F%3Fattachment_id%3D1433&psig=AOvVaw1H9CyEkJADk2Z10rcAj9IL&ust=1618666688271000&source=images&cd=vfe&ved=2ahUKEwiB6v248YLwAhWb7rsIHWbqCFYQjRx6BAgAEAc

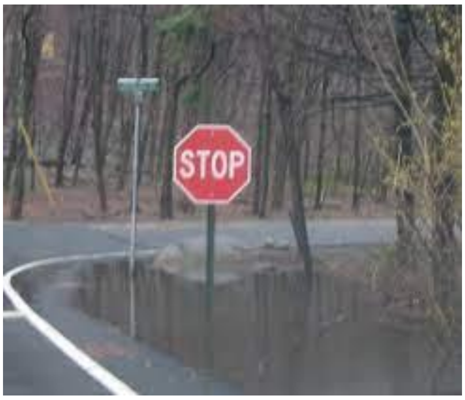

Mimage014

https://assets.nrdc.org/sites/default/files/styles/full_content/public/media-uploads/36766757522_13355b75b0_o_0.jpg?itok=R3TUxjtE

https://www.google.com/url?sa=i&url=https%3A%2F%2Fwww.nrdc.org%2Fexperts%2Fanna-weber%2Fbuyout-case-study-harris-county-texas&psig=AOvVaw0tpODH1xajZVadLO3fGreK&ust=1618666677463000&source=images&cd=vfe&ved=2ahUKEwiPkOqz8YLwAhV_g_0HHRgBBw8QjRx6BAgAEAc

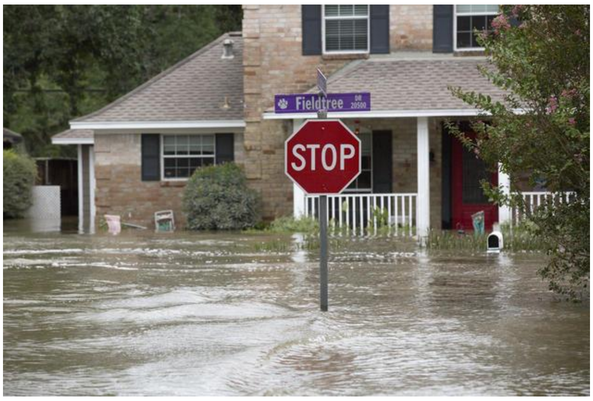

Mimage015

https://c8.alamy.com/comp/EEW71X/stop-sign-sticking-out-from-flood-waters-EEW71X.jpg

https://www.google.com/url?sa=i&url=https%3A%2F%2Fwww.alamy.com%2Fstock-photo-stop-sign-sticking-out-from-flood-waters-78000998.html&psig=AOvVaw1SAj2MtR9k67PKDEJjiaTh&ust=1618666663545000&source=images&cd=vfe&ved=2ahUKEwjo1Jit8YLwAhX8if0HHcaGAzgQjRx6BAgAEAc

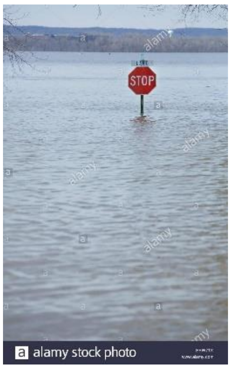

Mimage016

https://www.abc.net.au/news/image/8404598-3x2-940x627.jpg

https://www.google.com/url?sa=i&url=https%3A%2F%2Fwww.abc.net.au%2Fnews%2F2017-03-31%2Fstop-sign-peeks-out-of-lismore-floodwaters%2F8404796&psig=AOvVaw0NnnywesM0b7kZMNrdtY5K&ust=1618666652549000&source=images&cd=vfe&ved=2ahUKEwjkvvmn8YLwAhXQh_0HHc1gBz0QjRx6BAgAEAc

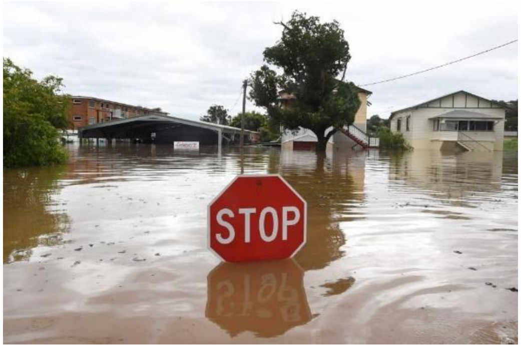

Mimage017

https://images.westend61.de/0001186686pw/flooding-around-stop-sign-and-streetlamp-BLEF02934.jpg

https://www.google.com/url?sa=i&url=https%3A%2F%2Fwww.westend61.de%2Fen%2FimageView%2FBLEF02934%2Fflooding-around-stop-sign-and-streetlamp&psig=AOvVaw0VgrKwQkPX7PFI9GVg_Q7d&ust=1618666646413000&source=images&cd=vfe&ved=2ahUKEwjpg4Ol8YLwAhXRhP0HHe01Ai4QjRx6BAgAEAc

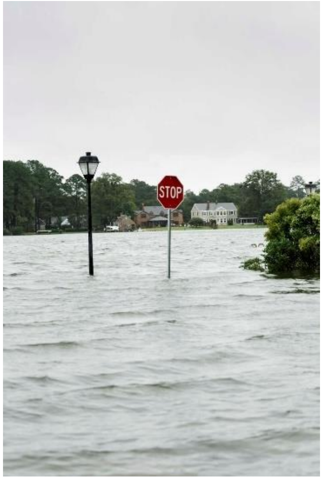

Mimage018

https://img.apmcdn.org/80c10ce735aeb1b4557c842ecb55f4282c1f3755/normal/3e0d07-20100323-flooding-in-moorhead.jpg

https://www.google.com/url?sa=i&url=https%3A%2F%2Fwww.mprnews.org%2Fstory%2F2011%2F03%2F23%2Fflood-2011-resources-links&psig=AOvVaw1p3XDjEi9w70dKqhXeyiWQ&ust=1618666639763000&source=images&cd=vfe&ved=2ahUKEwilI-2h8YLwAhWQyrsIHekeDeYQjRx6BAgAEAc

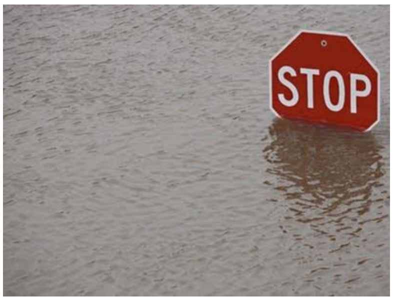

Mimage019

https://bloximages.newyork1.vip.townnews.com/stltoday.com/content/tncms/assets/v3/editorial/9/90/990eeef4-8454-5a72-90ca-4e45a0f16e28/5c72dc1c4c8e7.image.jpg?resize=1200%2C830

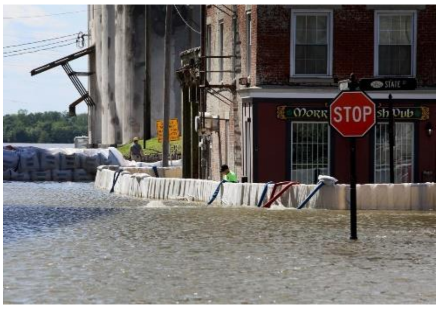

Mimage020

https://www.mlive.com/resizer/6P48dAen5f2g8S-PHH9gaCwHd0M=/450x0/smart/arc-anglerfish-arc2-prod-advancelocal.s3.amazonaws.com/public/R3PSNANJD5DYBKW4FR3JXGGIUQ.JPG

https://www.google.com/url?sa=i&url=https%3A%2F%2Fwww.mlive.com%2Fnews%2Fsaginaw-bay-city%2F2020%2F05%2Fwhat-you-need-to-know-about-the-midland-flood-thursday-may-21.html&psig=AOvVaw00sD4obottjeh71rMEvPxT&ust=1618936175253000&source=images&cd=vfe&ved=2ahUKEwil7LGu3YrwAhVc4bsIHfAAAqoQjRx6BAgAEAc

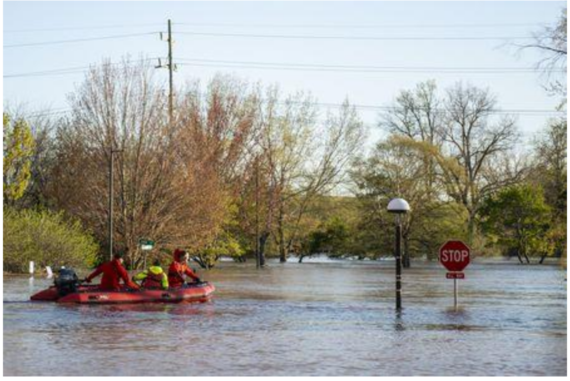

Mimage021

https://newscdn.weigelbroadcasting.com/acDNH-1573751458-embed-sb%20flooding.jpg

https://www.google.com/url?sa=i&url=https%3A%2F%2Fwww.abc57.com%2Fnews%2FSouth-Bend-working-to-mitigate-impacts-of-climate-change-with-new-plan&psig=AOvVaw18aty6gywDJGTMUCJ_Yhzt&ust=1618936177583000&source=images&cd=vfe&ved=2ahUKEwiskcCv3YrwAhVTROUKHUItAlwQjRx6BAgAEAc

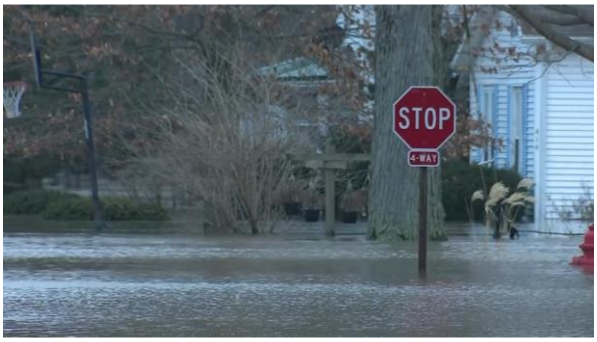

Mimage022

https://i.insider.com/5c6c4ad20d15f548bd4d3093?width=700

https://www.google.com/url?sa=i&url=https%3A%2F%2Fwww.businessinsider.com%2Fflooding-sea-level-rise-cost-town-100000s-in-lost-income-2019-2&psig=AOvVaw2CMK1Au46AuQm1lXgV55Ae&ust=1618936182398000&source=images&cd=vfe&ved=2ahUKEwj29uWx3YrwAhUXh_0HHX37BlIQjRx6BAgAEAc

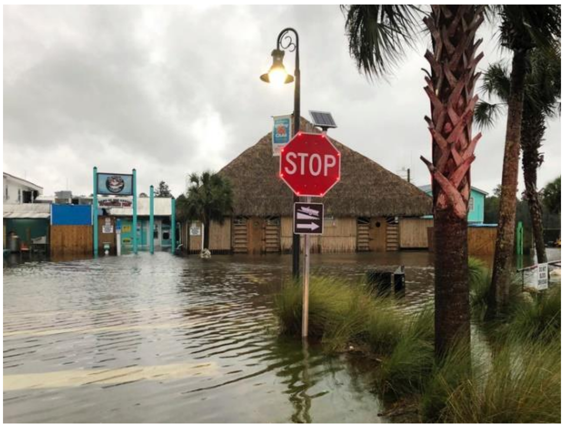

Mimage023

https://cdn.cnn.com/cnnnext/dam/assets/210309095755-04-kaupakalua-dam-flooding-0308-exlarge-169.jpg

https://www.google.com/url?sa=i&url=https%3A%2F%2Fwww.cnn.com%2F2021%2F03%2F10%2Fus%2Fmaui-hawaii-flooding-wednesday%2Findex.html&psig=AOvVaw3ze6AGgocjpvfnPeUgysWZ&ust=1618936206589000&source=images&cd=vfe&ved=2ahUKEwjopqq93YrwAhXp47sIHWutA2sQjRx6BAgAEAc

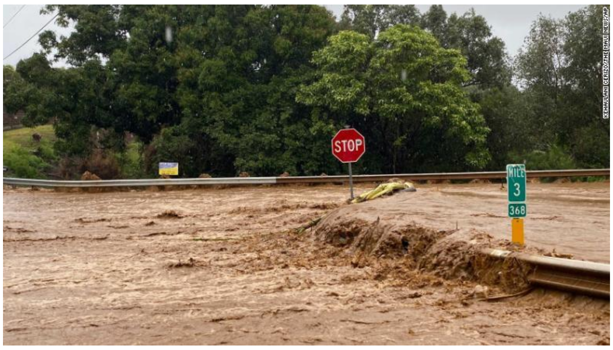

Mimage025

https://www.mercurynews.com/wp-content/uploads/2019/02/SJM-L-GUERNEVILLE-0228-66.jpg?w=620

https://www.google.com/url?sa=i&url=https%3A%2F%2Fwww.mercurynews.com%2F2019%2F02%2F27%2Fthis-sonoma-county-town-got-20-inches-of-rain-in-48-hours-san-jose-averages-about-15-a-year&psig=AOvVaw3nuUTEgX44wBU6THFBOtk1&ust=1618936218723000&source=images&cd=vfe&ved=2ahUKEwi7g4_D3YrwAhXWh_0HHYQ-BJkQjRx6BAgAEAc

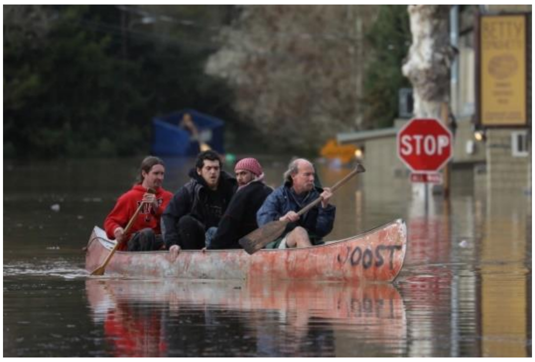

Mimage026

https://cdn.slidesharecdn.com/ss_thumbnails/floodsafety-100407133226-phpapp01-thumbnail-4.jpg?cb=1270808746

https://www.google.com/url?sa=i&url=https%3A%2F%2Fwww.slideshare.net%2Ffairfaxcounty%2Fflood-safety&psig=AOvVaw09IhsNxtjkjsDS0N21EdcK&ust=1618936221392000&source=images&cd=vfe&ved=2ahUKEwiM-rHE3YrwAhXehP0HHY5-CusQjRx6BAgAEAc

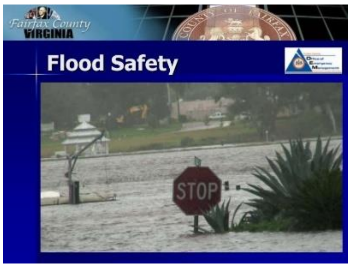

Mimage027

https://s.hdnux.com/photos/01/12/10/46/19433919/3/375x250.jpg

https://www.google.com/url?sa=i&url=https%3A%2F%2Fwww.ourmidland.com%2Fnews%2Farticle%2FPhotos-Downtown-Midland-flood-level-Wednesday-15282731.php&psig=AOvVaw0SnKSCueh26UXY2g6J2l_0&ust=1618936223000000&source=images&cd=vfe&ved=2ahUKEwj6kZTF3YrwAhUk5rsIHdt8BLcQjRx6BAgAEAc

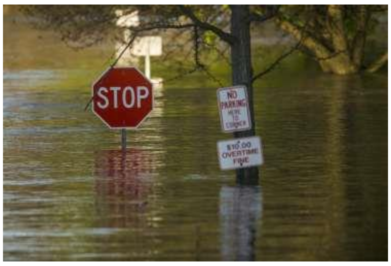

Mimage028

https://thumbs.dreamstime.com/b/stop-sign-flood-hochwasser-concept-image-flooded-intersection-31517399.jpg

https://www.google.com/url?sa=i&url=https%3A%2F%2Fwww.dreamstime.com%2Fillustration%2Fhochwasser.html&psig=AOvVaw3LUDJ-_GML1NIsWzgxa3uX&ust=1618936230797000&source=images&cd=vfe&ved=2ahUKEwjgifDI3YrwAhV64bsIHZ4PBgAQjRx6BAgAEAc

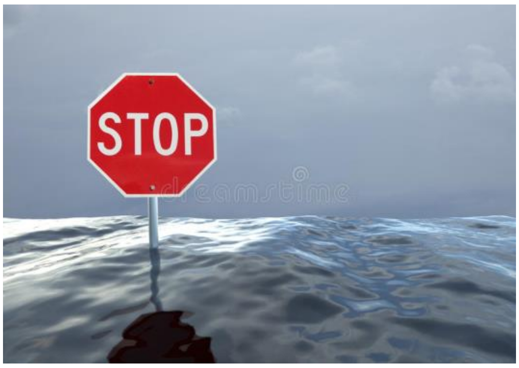

Mimage029

https://www.gannett-cdn.com/presto/2020/10/02/PTCN/7c36d86e-7911-4360-ae1f-6b8d2b1a814c-1b1b4d61-d260-4dd9-96ac-1b7143a0e706_thumbnail.png?width=1280&height=720&fit=crop&format=pjpg&auto=webp

https://www.google.com/url?sa=i&url=https%3A%2F%2Fwww.tcpalm.com%2Fstory%2Fweather%2F2020%2F10%2F02%2Fflooded-roads-impact-treasure-coast-cooler-temperatures-forecast%2F5893238002%2F&psig=AOvVaw2hR5thKNQ4ywMwmhtdbKDB&ust=1618936245345000&source=images&cd=vfe&ved=2ahUKEwix9OfP3YrwAhXriv0HHdX-DzEQjRx6BAgAEAc

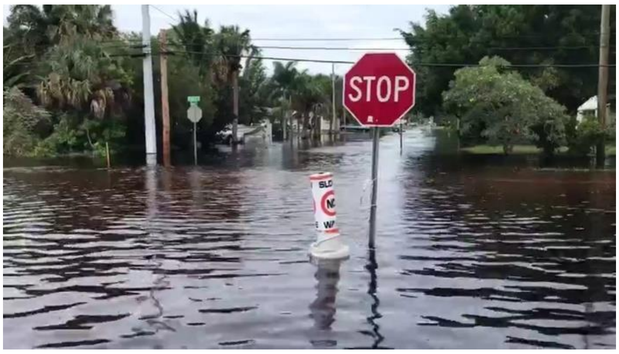

Mimage030

https://www.nj.com/resizer/jO_REUM0IGIlsLVHztYv4EtPQVc=/1280x0/smart/advancelocal-adapter-image-uploads.s3.amazonaws.com/image.nj.com/home/njo-media/width2048/img/ledgerlocal/photo/sayreville-flood-2007jpg-6bb47c1b6ce129b8.jpg

https://www.google.com/url?sa=i&url=https%3A%2F%2Fwww.nj.com%2Fnews%2Flocal%2F2010%2F05%2Fsayreville_flood_victims_still_1.html&psig=AOvVaw3RjyP-MdqBhKniCjbtQuN_&ust=1618936258533000&source=images&cd=vfe&ved=2ahUKEwii7ozW3YrwAhWG4bsIHTXyAJ0QjRx6BAgAE

Ac

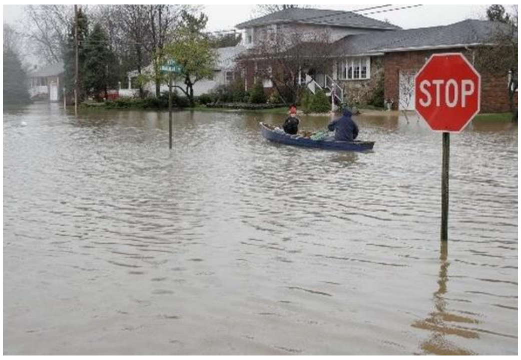

Mimage031

https://flooddefenders.org/hubfs/escambiastop.jpg

https://www.google.com/url?sa=i&url=https%3A%2F%2Fflooddefenders.org%2Fnews&psig=AOvVaw2Wt1PH7xfo7HvEyuF2Ej8p&ust=1618936261436000&source=images&cd=vfe&ved=2ahUKEwjhg77X3YrwAhWk47sIHZ25A8cQjRx6BAgAEAc

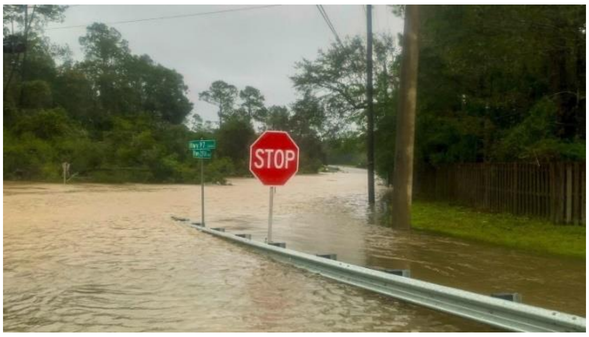

Mimage032

https://www.gannett-cdn.com/-mm-/e0afd45027b74bed9cc3ffd406c9b07636faee8f/c=0-113-4395-2596/local/-/media/2018/02/26/Louisville/Louisville/636552513666563815-UticaFloodingMonday-6.JPG

https://www.google.com/url?sa=i&url=https%3A%2F%2Fwww.courier-journal.com%2Fstory%2Fnews%2Flocal%2F2018%2F02%2F28%2Fjoin-courier-journal-facebook-conversation-flooding%2F381081002%2F&psig=AOvVaw2NT1vyV_JpDFojXMLeenO7&ust=1618936265354000&source=images&cd=vfe&ved=2ahUKEwiZlq3Z3YrwAhUpybsIHRazCuUQjRx6BAgAEAc

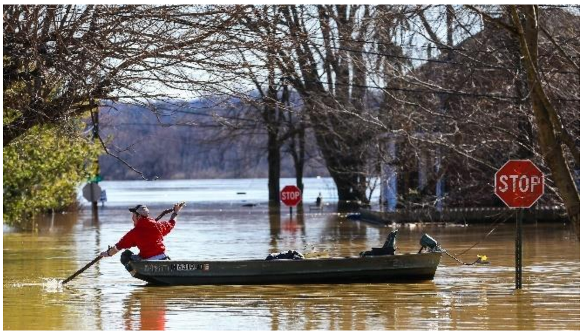

Mimage033

https://bloximages.chicago2.vip.townnews.com/enidnews.com/content/tncms/assets/v3/editorial/b/81/b81da7ec-d137-11ea-811e-e350f7aee62f/5f20cb8f13023.image.jpg?resize=1200%2C800

https://www.google.com/url?sa=i&url=https%3A%2F%2Fwww.enidnews.com%2Fnews%2Fheavy-rain-causes-street-flooding%2Farticle_da92bdae-d136-11ea-b35c-df9608d7f800.html&psig=AOvVaw06SgIG88zc_HbftcNrFLRH&ust=1618936270825000&source=images&cd=vfe&ved=2ahUKEwifmfvb3YrwAhW8wLsIHfgRDjAQjRx6BAgAEAc

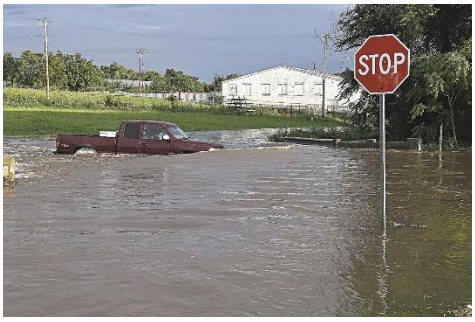

Mimage034

https://www.wafb.com/resizer/e0UtGqS6KD1TeOO6eFKJD77ZSNo=/1200x600/arc-anglerfish-arc2-prod-raycom.s3.amazonaws.com/public/KGTQIMOL4RA2NOPDVZKXKSPAVQ.jpg

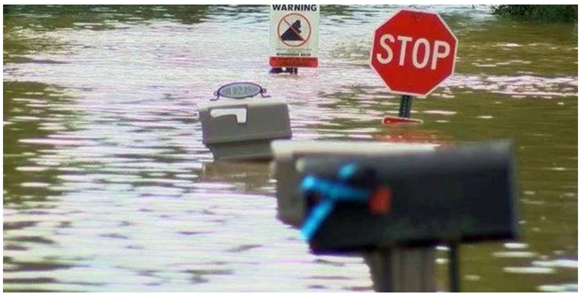

Mimage035

https://www.google.com/url?sa=i&url=https%3A%2F%2Fwww.facebook.com%2Fpages%2FFlood-Control-Movement-Get-Involved%2F386524755057450&psig=AOvVaw3G2vVwHSBIKyfdOTAeIZ-y&ust=1618936313355000&source=images&cd=vfe&ved=2ahUKEwj6-p7w3YrwAhVhgv0HHb_LDXIQjRx6BAgAEAc

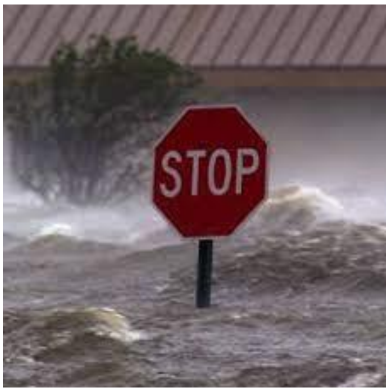

Mimage036

https://bloximages.newyork1.vip.townnews.com/foxcarolina.com/content/tncms/assets/v3/editorial/a/e4/ae455bea-9a2b-11ea-9072-eb5499efa9e9/5ec4710a9b519.image.jpg?resize=1200%2C1600

https://www.google.com/url?sa=i&url=https%3A%2F%2Fwww.foxcarolina.com%2Fnews%2Fflood-warning-issued-as-lake-lure-gates-raised-due-to-rainfall%2Farticle_5986535e-9a24-11ea-a9ae-cb871c1a5647.html&psig=AOvVaw2-yrCvo1DkEVnesgSqTqGh&ust=1618936317147000&source=images&cd=vfe&ved=2ahUKEwiJtoby3YrwAhWkTOUKHQlgDPcQjRx6BAgAEAc

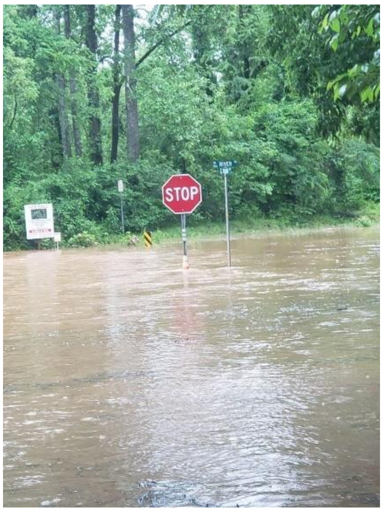

Mimage037

https://news.clemson.edu/wp-content/uploads/2018/11/LIHTC-flooding-pix.jpg

https://www.google.com/url?sa=i&url=https%3A%2F%2Fnews.clemson.edu%2Fflorida-tax-credit-housing-is-often-in-flood-zones-study-finds%2F&psig=AOvVaw2PoK3BVqgk27wiva_Vv12M&ust=1618936319395000&source=images&cd=vfe&ved=2ahUKEwjY0Y_z3YrwAhVryLsIHbAPCPAQjRx6BAgAEAc

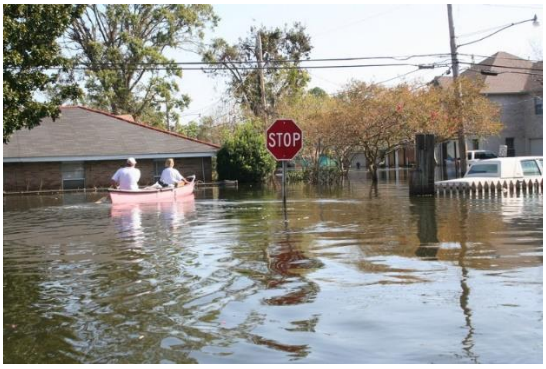

Mimage038

https://ewscripps.brightspotcdn.com/dims4/default/0c14d0b/2147483647/strip/true/crop/640x427+0+0/resize/640x427!/quality/90/?url=https%3A%2F%2Fsharing.kgun9.com%2Fsharescnn%2Fphoto%2F2018%2F03%2F02%2FGettyImages-926386668_1520035734361_79845066_ver1.0_640_480.jpg

https://www.google.com/url?sa=i&url=https%3A%2F%2Fwww.kgun9.com%2Fnews%2Fbomb-cyclone-pounds-east-coast-with-heavy-flooding-high-winds&psig=AOvVaw35QBBsgNxbc4vp4QOLoL8d&ust=1618936330354000&source=images&cd=vfe&ved=2ahUKEwiEvKz43YrwAhW37LsIHcTSDWsQjRx6BAgAEAc

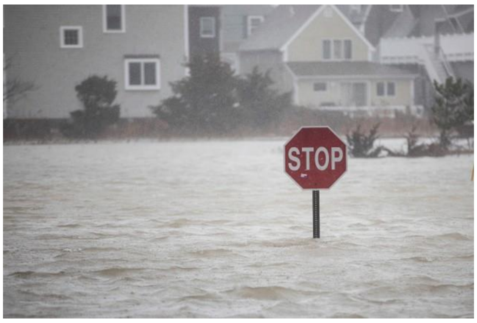

Mimage039

https://cbs12.com/resources/media2/16x9/full/1015/center/80/3049393e-000b-4acc-8cb0-8087e6367fec-large16x9_flood.jpg

https://www.google.com/url?sa=i&url=https%3A%2F%2Fcbs12.com%2Fnews%2Flocal%2Fflooding-plagues-local-neighborhood&psig=AOvVaw1xX1u562m0dDtNLdaOAh-r&ust=1618936333856000&source=images&cd=vfe&ved=2ahUKEwiVnYL63YrwAhWm47sIHbCgACcQjRx6BAgAEAc

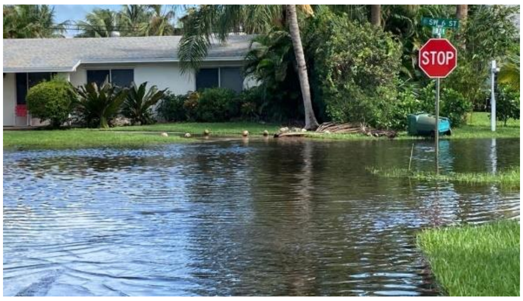

Mimage040

https://www.gnsnews.co.in/wp-content/uploads/2019/03/flood.jpg

https://www.google.com/url?sa=i&url=https%3A%2F%2Fwww.gnsnews.co.in%2Fbomb-cyclone-flood-parts-of-nebraska-and-u-s-central-plains-were-underwater%2F&psig=AOvVaw2hv2zCoR8a2GO5xsnDDS6i&ust=1618936351395000&source=images&cd=vfe&ved=2ahUKEwiN2bCC3orwAhXlxLsIHc49Bf8QjRx6BAgAEAc

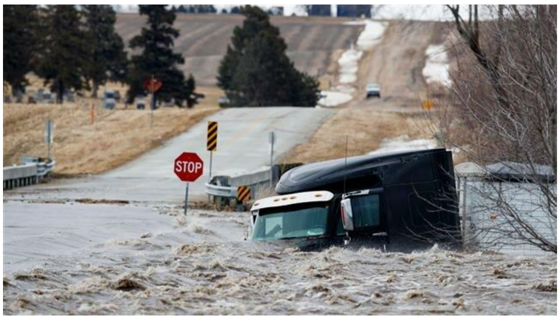

Mimage041

https://m.salisburypost.com/wp-content/uploads/sites/9/2020/08/Flooding-rain-JB4-1.jpg?w=526

https://www.google.com/url?sa=i&url=https%3A%2F%2Fwww.salisburypost.com%2F2020%2F08%2F31%2Fflash-flooding-submerges-vehicles-surrounds-homes-off-of-jake-alexander-

boulevard%2F&psig=AOvVaw1YV6DoLjEEdEczp3DwetOD&ust=1618937804949000&source=images&cd=vfe&ved=2ahUKEwiq0r6344rwAhVT7LsIHZzhDjMQjRx6BAgAEAc

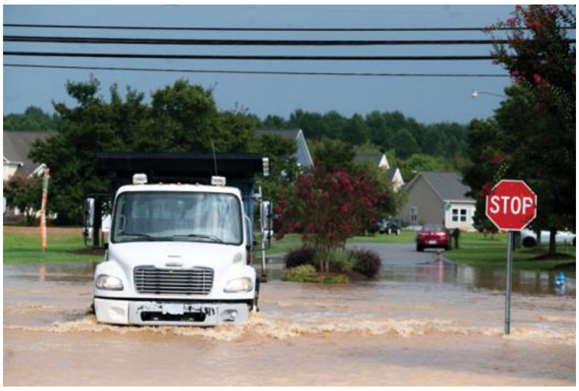

Mimage042

https://serc.carleton.edu/download/images/48007/flooded_street_sign_moorhead.v6.jpg

https://www.google.com/url?sa=i&url=https%3A%2F%2Fserc.carleton.edu%2Fdetails%2Fimages%2F48007.html&psig=AOvVaw2ecO9BeAv9ndm20pptYl_A&ust=1618937808262000&source=images&cd=vfe&ved=2ahUKEwid64i544rwAhV45rsIHTPPBncQjRx6BAgAEAc

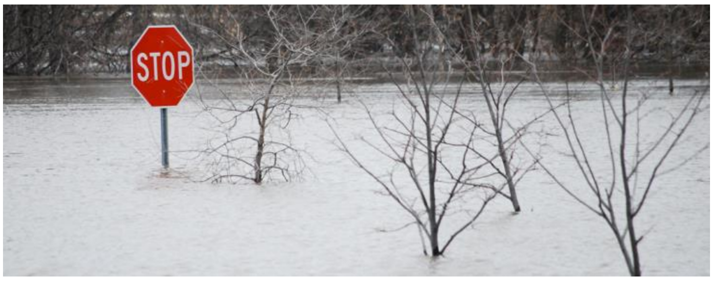

Mimage043

https://media.npr.org/assets/img/2011/06/27/117449586_7802901_wide-403c7d44c67dcd4fe44a4039499690c45c187a32.jpg?s=1400

https://www.google.com/url?sa=i&url=https%3A%2F%2Fwww.npr.org%2Fsections%2Fthetwo-way%2F2011%2F06%2F27%2F137442969%2Fbachman-launches-campaign-flood-waters-recede-in-minot&psig=AOvVaw3qS7OrUsWNtkbxisLVtkAq&ust=1618937814882000&source=images&cd=vfe&ved=2ahUKEwip85y844rwAhWq5LsIHd99CMAQjRx6BAgAEAc

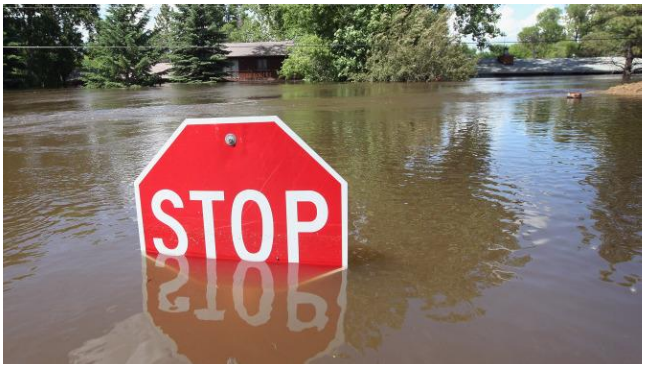

Mimage045

http://www.blufftonicon.com/news/2015/06/16/flooding-bluffton-heres-what-we-know-11-am-northbound-i-75-closed-bentley-road

https://www.google.com/url?sa=i&url=http%3A%2F%2Fwww.blufftonicon.com%2Fnews%2F2015%2F06%2F16%2Fflooding-bluffton-heres-what-we-know-11-am-northbound-i-75-closed-bentley-road&psig=AOvVaw3h9Fs20F2VSs79rAcBVlvb&ust=1618937832579000&source=images&cd=vfe&ved=2ahUKEwiGhtXE44rwAhVC7rsIHRjwDVcQjRx6BAgAEAc

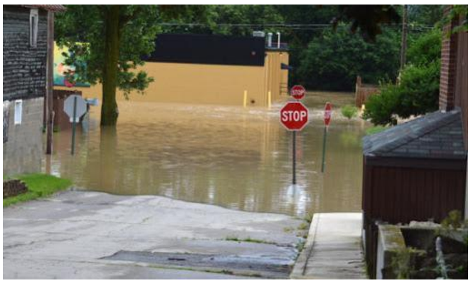

Mimage046

https://i.insider.com/5c77f9c126289867bc72f699?width=1000&format=jpeg&auto=webp

https://www.google.com/url?sa=i&url=https%3A%2F%2Fwww.businessinsider.com%2Fcalifornia-flooding-photos-wine-country-2019-2&psig=AOvVaw3o6qyF7ag5J6i5EGKDWfzN&ust=1618937842875000&source=images&cd=vfe&ved=2ahUKEwiqvsnJ44rwAhX-8rsIHTjsA1wQjRx6BAgAEAc

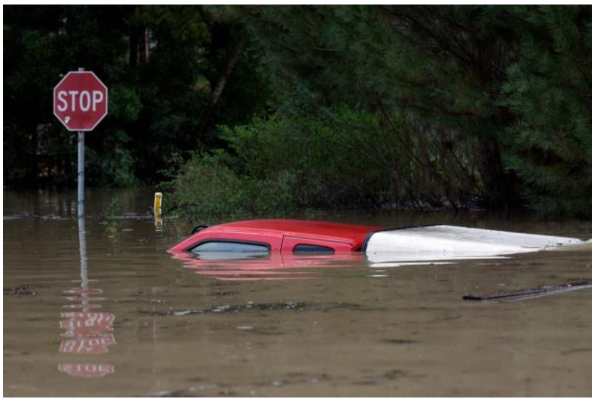

Mimage047

https://providentliving.churchofjesuschrist.org/bc/content/providentliving/content/images/flood-damage-cleanup_1294542_480x270.jpg

https://www.google.com/url?sa=i&url=https%3A%2F%2Fprovidentliving.churchofjesuschrist.org%2Fleader%2Femergency-preparedness-and-response%2Farea-planning-guide%2Fflood-damage-cleanup-items%3Flang%3Deng&psig=AOvVaw2SU-uHX9TgynAoKnaB-Rj4&ust=1618937851051000&source=images&cd=vfe&ved=2ahUKEwjJubzN44rwAhXM0bsIHY3FBaAQjRx6BAgAEAc

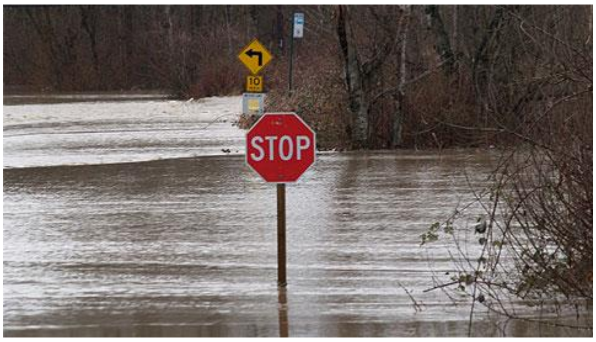

Mimage048

https://www.google.com/url?sa=i&url=https%3A%2F%2Fwww.wapt.com%2Farticle%2Fflooding-from-approaching-storms-real-concern-for-emergency-officials%2F30459557&psig=AOvVaw2rQ4ZjtcRv3mIO7MIVlVrO&ust=1618937854023000&source=images&cd=vfe&ved=2ahUKEwjL8fHO44rwAhWFROUKHbwgD30QjRx6BAgAEAc

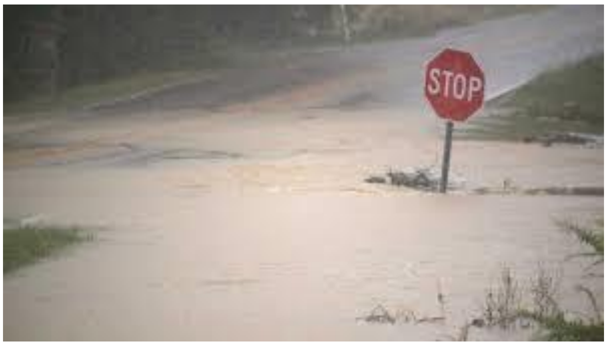

Mimage049

https://canadawater.files.wordpress.com/2011/04/flooding-in-manitoba-oct-2010.jpg?w=584

https://www.google.com/url?sa=i&url=https%3A%2F%2Fsavelakewinnipeg.org%2F2011%2F04%2F13%2Fspring-floods-across-manitoba-saskatchewan-and-north-dakota-%25E2%2580%2593-road-closures-paint-a-striking-picture%2F&psig=AOvVaw0DxHA7pTV-ZhAmtk_Rou-k&ust=1618937856125000&source=images&cd=vfe&ved=2ahUKEwjHkfLP44rwAhUy67sIHTmgAYYQjRx6BAgAEAc

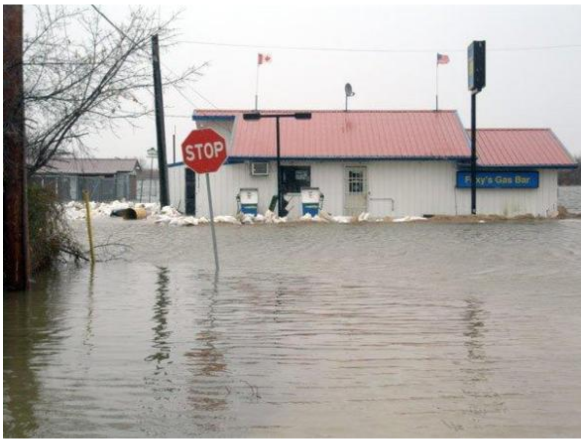

Mimage050

https://st.focusedcollection.com/14144030/i/650/focused_179969070-stock-photo-flooded-street-stop-road-sign.jpg

https://www.google.com/url?sa=i&url=https%3A%2F%2Ffocusedcollection.com%2F179969070%2Fstock-photo-flooded-street-stop-

road-sign.html&psig=AOvVaw1ZIl8H7YwusXKPIohM4T6U&ust=1618666835050000&source=images&cd=vfe&ved=2ahUKEwiNyvz-8YLwAhVv6rsIHbjSAzcQjRx6BAgAEAc

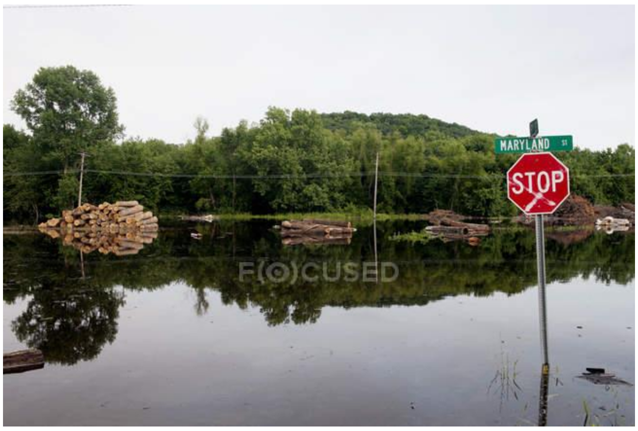

Mimage051

https://www.google.com/url?sa=i&url=https%3A%2F%2Fweather.com%2Fstorms%2Fsevere%2Fnews%2F2018-02-22-tornado-flash-flood-warning-action&psig=AOvVaw2u6J4119e-IAkHL-tqMMd8&ust=1618937870235000&source=images&cd=vfe&ved=2ahUKEwj_rc_W44rwAhW847sIHfE1A0YQjRx6BAgAEAc

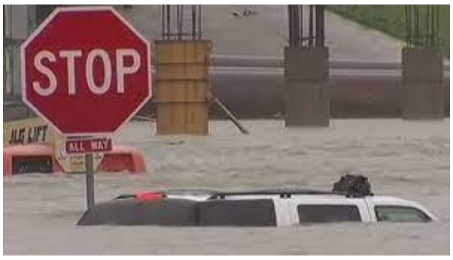

Mimage052

https://static.seattletimes.com/wp-content/uploads/2019/02/urn-publicid-ap-org-a11fe39e85674c0d8c1b6d863c56d81aDeep_South_Weather_79591-780x433.jpg

https://www.google.com/url?sa=i&url=https%3A%2F%2Fwww.seattletimes.com%2Fnation-world%2Fnation%2Fwaters-start-to-slowly-recede-in-parts-of-flooded-south%2F&psig=AOvVaw3P4ztJs-F9UOtLBu6w50Fn&ust=1618937873450000&source=images&cd=vfe&ved=2ahUKEwiYyJPY44rwAhVlyrsIHfsEC8kQjRx6BAgAEAc

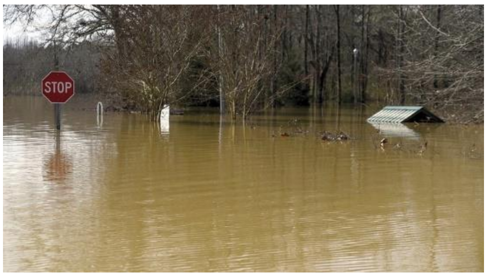

Mimage053

https://smartcdn.prod.postmedia.digital/windsorstar/wp-content/uploads/2018/02/flood-03-jpg.jpg

https://www.google.com/url?sa=i&url=https%3A%2F%2Fwindsorstar.com%2Fnews%2Flocal-news%2Fwater-levels-still-high-but-worst-is-over-in-chatham-kent-flooding&psig=AOvVaw0SBOQivbpSiWeWr7tNLrv8&ust=1618666872718000&source=images&cd=vfe&ved=2ahUKEwiz0feQ8oLwAhUT47sIHVn0BMYQjRx6BAgAEAc

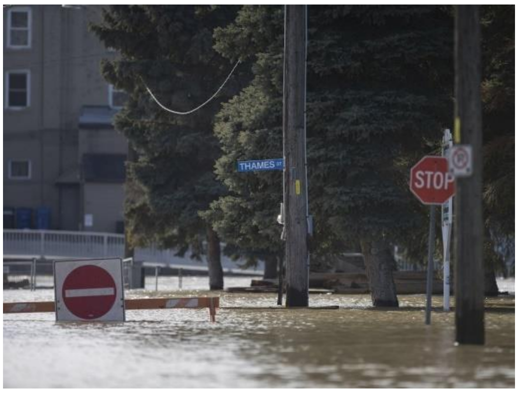

Mimage054

https://cloudfront-us-east-1.images.arcpublishing.com/pmn/4N3YNCVSMRE4RLLIU4P3S6VPPQ.jpg

https://www.google.com/url?sa=i&url=https%3A%2F%2Fwww.inquirer.com%2Fweather%2Ftropical-storm-warning-fay-jersey-shore-flood-watch-20200710.html&psig=AOvVaw14UWUUDbuh7RrLRks-caqx&ust=1618937879102000&source=images&cd=vfe&ved=2ahUKEwiMy-za44rwAhWB4bsIHaQACFQQjRx6BAgAEAc

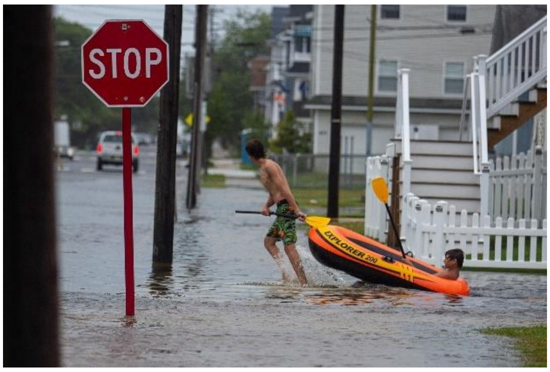

Mimage055

https://imengine.prod.srp.navigacloud.com/?uuid=83598442-4423-43D6-AE9D-45F8FE9EECF8&type=primary&q=72&width=1200

https://www.google.com/url?sa=i&url=https%3A%2F%2Fwww.pressdemocrat.com%2Farticle%2Fnews%2Fsonoma-county-flood-submerges-the-barlow-shopping-district-in-sebastopol%2F&psig=AOvVaw15DVY2FN-tnFeEaYQ1BusY&ust=1618937881029000&source=images&cd=vfe&ved=2ahUKEwjRmeLb44rwAhUVxrsIHWaZAWwQjRx6BAgAEAc

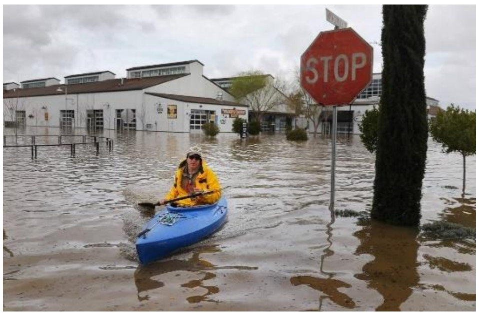

Mimage056

https://bloximages.newyork1.vip.townnews.com/williamsonhomepage.com/content/tncms/assets/v3/editorial/6/73/673fffe0-8bf6-11ea-bb37-e3b43044870b/5eac9ab3d4423.image.jpg

https://www.google.com/url?sa=i&url=https%3A%2F%2Fwww.williamsonhomepage.com%2Fbrentwood%2F10-years-after-the-flood-were-still-thankful-for-your-readership%2Farticle_3ba1b87c-8bda-11ea-b629-ef3718cbadc3.html&psig=AOvVaw0wss_8PKM0gjuC0MBJ8C42&ust=1618937889563000&source=images&cd=vfe&ved=2ahUKEwiniuvf44rwAhVT7LsIHZzhDjMQjRx6BAgAEAc

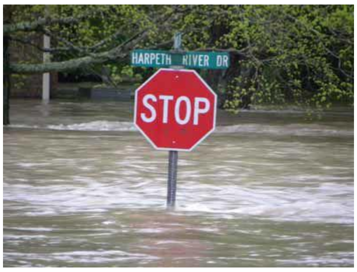

Mimage057

https://www.click2houston.com/resizer/aWdnsoHaJwMAwlfhm8YijJKIzAQ=/fit-in/640x360/smart/filters:format(jpeg):strip_exif(true):strip_icc(true):no_upscale(true):quality(65):fill(FFF)/cloudfront-us-east-1.images.arcpublishing.com/gmg/M5JUCYNMNZBVVKV6A2CJ5PLECI.JPG

https://www.google.com/url?sa=i&url=https%3A%2F%2Fwww.click2houston.com%2Fweather%2F2020%2F05%2F11%2Fwhy-we-have-to-watch-for-flooding-this-weekend%2F&psig=AOvVaw1LyVEshkriZN_G_MQUfRiC&ust=1618937900376000&source=images&cd=vfe&ved=2ahUKEwi8if_k44rwAhUshP0HHR1ADXIQjRx6BAgAEAc

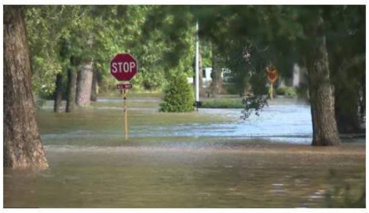

Mimage058

https://cbs12.com/resources/media/db6e91ed-5c02-4af8-8fce-e98a89e7a969-medium16x9_thumb_86407.png?1601694773141

https://www.google.com/url?sa=i&url=https%3A%2F%2Fcbs12.com%2Fnews%2Flocal%2Fhobe-sound-neighborhood-continues-to-flood&psig=AOvVaw16akJPNlUprMzjSH5Qk03v&ust=1618937903621000&source=images&cd=vfe&ved=2ahUKEwj1kMXm44rwAhW

i5bsIHfKkATgQjRx6BAgAEAc

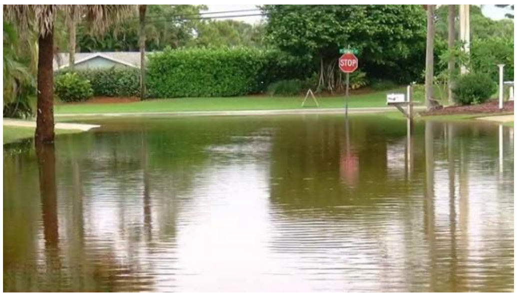

Mimage059

https://bloximages.newyork1.vip.townnews.com/postandcourier.com/content/tncms/assets/v3/editorial/2/0e/20ebf3d0-6a94-11e8-aa3e-372777c4ae35/59b5911a61856.image.jpg?resize=1200%2C827

https://www.google.com/url?sa=i&url=https%3A%2F%2Fwww.postandcourier.com%2Fnews%2Fsun-setting-on-charlestons-notorious-sunny-day-flooding-spots%2Farticle_806d7a10-6a88-11e8-adf6-637efe334755.html&psig=AOvVaw3LzV1m2FnjaGKUxqE7wTzu&ust=1618937909912000&source=images&cd=vfe&ved=2ahUKEwi2hcXp44rwAhU94bsIHeWjDMYQjRx6BAgAEAc

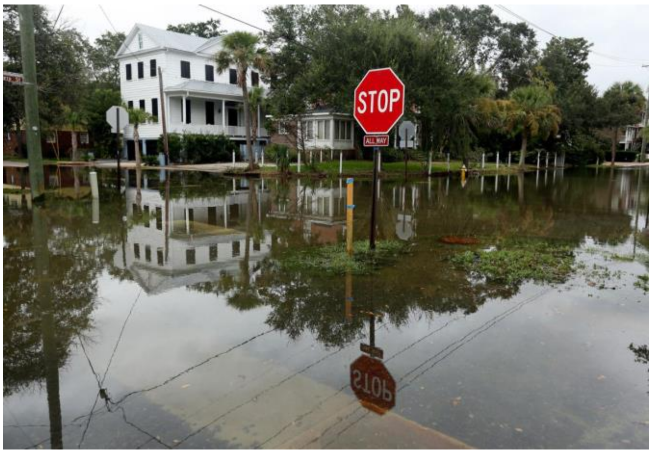

Mimage060

https://seaislenews.com/wp-content/uploads/sites/3/2017/06/1.4-no-wake-flooding-central-and-42nd-800x450.jpg

https://www.google.com/url?sa=i&url=https%3A%2F%2Fseaislenews.com%2Fsea-isle-looks-to-crack-down-on-trucks-speeding-through-flooded-areas%2F&psig=AOvVaw3eb3Ok_OD8jFzrP6LgHHKx&ust=1618937912705000&source=images&cd=vfe&ved=2ahUKEwjmxO_q44rwAhVX5bsIHXVKDB4QjRx6BAgAEAc

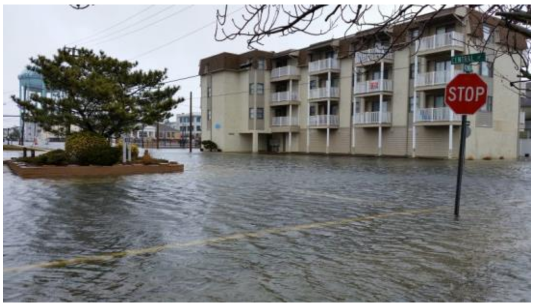

Mimage061

https://news.wsiu.org/sites/wsiu/files/styles/x_large/public/201903/ohio_flood1.png

https://www.google.com/url?sa=i&url=https%3A%2F%2Fnews.wsiu.org%2Fpost%2Fanother-year-winter-flooding-lower-ohio-valley&psig=AOvVaw36pAbsFL_AM1rN7stoeXsq&ust=1618937915416000&source=images&cd=vfe&ved=2ahUKEwjL_ZTs44rwAhXO67sIHUw6BbMQjRx6BAgAEAc

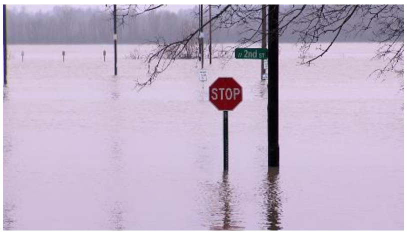

Mimage062

https://www.gannett-cdn.com/-mm-/1a7c0fb192c1aa67803f26c16a70beb3b8d6e9e0/c=16-0-4223-2377/local/-/media/2015/06/06/LAGroup/Shreveport/635691978199346144-SHR-flooding-0607-17.JPG

https://www.google.com/url?sa=i&url=https%3A%2F%2Fwww.shreveporttimes.com%2Fstory%2Fnews%2Flocal%2F2015%2F06%2F06%2Fbossier-sheriff-latest-road-closures-areas-impacted--flooding%2F28609327%2F&psig=AOvVaw0b_pQDHrWaj8-8lROWhac-&ust=1618937920216000&source=images&cd=vfe&ved=2ahUKEwi_-Lnu44rwAhXM0bsIHY3FBaAQjRx6BAgAEAc

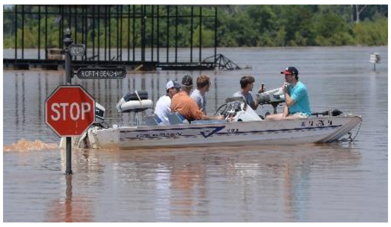

Mimage063

https://bloximages.newyork1.vip.townnews.com/qconline.com/content/tncms/assets/v3/editorial/4/36/43617a2a-8e40-5ad4-ae7e-fb300f56f24d/5c94fe4ad34d2.image.jpg?resize=1200%2C888

https://www.google.com/url?sa=i&url=https%3A%2F%2Fqconline.com%2Fnews%2Flocal%2Fmississippi-river-exceeding-major-flood-stage-but-bigger-flood-could-be-on-the-way%2Farticle_ee77813e-33a2-5f25-83ba-651f37868ae8.html&psig=AOvVaw0oe2FRY513Pz56YuH08Pdk&ust=1618937923957000&source=images&cd=vfe&ved=2ahUKEwjfo57w44rwAhUOgf0HHaasBiMQjRx6BAgAEAc

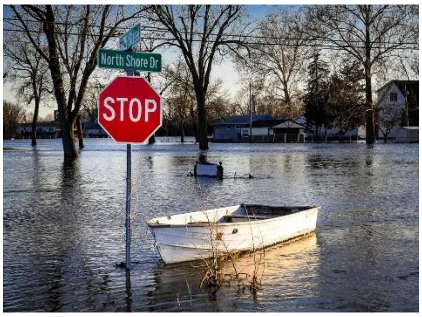

Mimage064

https://c8.alamy.com/comp/BWN2J5/usa-illinois-stop-sign-in-flood-BWN2J5.jpg

https://www.google.com/url?sa=i&url=https%3A%2F%2Fwww.alamy.com%2Fstock-photo%2Ftext-flood.html&psig=AOvVaw2GVT0ygbjzCQZZnHsLp4b5&ust=1618937927231000&source=images&cd=vfe&ved=2ahUKEwjUk-bx44rwAhUy67sIHTmgAYYQjRx6BAgAEAc

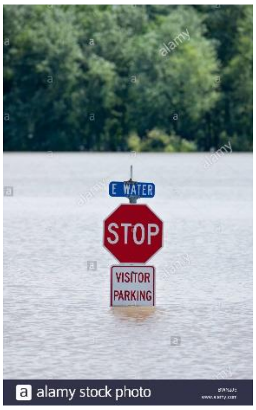

Mimage065

https://static01.nyt.com/newsgraphics/2017/09/01/flood-damaged-homes/436ef4ef43e4bdfb584070572b4aca13c892b703/841334410.jpg

https://www.google.com/url?sa=i&url=https%3A%2F%2Fwww.nytimes.com%2Finteractive%2F2017%2F09%2F01%2Fus%2Fhouston-damaged-buildings-in-fema-flood-zones.html&psig=AOvVaw0LAffXk2SVuZXX_XQzF1ng&ust=1618937934409000&source=images&cd=vfe&ved=2ahUKEwiPoJz144rwAhWM4bsIHVZaDCcQjRx6BAgAEAc

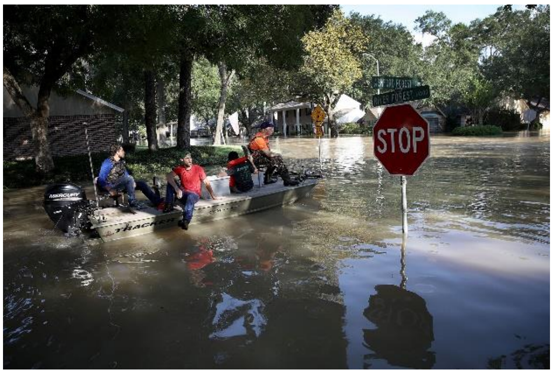

Mimage066

https://content.api.news/v3/images/bin/947fce27a7403a44de43afebb4337bda

https://www.google.com/url?sa=i&url=https%3A%2F%2Fwww.adelaidenow.com.au%2Fnews%2Fnational%2Froadways-flood-as-severe-storms-sweep-through-central-illinois%2Fvideo%2F21f7c604b678c8b73a377b60c198513d&psig=AOvVaw0smDKHGc4ggMcheg78RPkA&ust=1618936303969000&source=images&cd=vfe&ved=2ahUKEwiIiuLr3YrwAhVc4bsIHfAAAqoQjRx6BAgAEAc

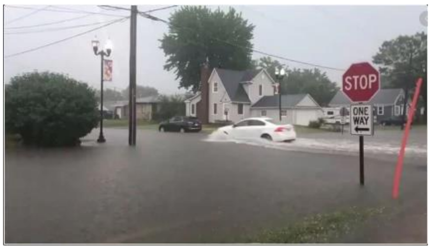

Mimage067

https://images.pond5.com/flood-water-reaches-level-street-footage-081374488_iconl.jpeg

https://www.google.com/url?sa=i&url=https%3A%2F%2Fwww.pond5.com%2Fstock-footage%2Fitem%2F81374488-flood-water-
reaches-level-street-sign-during-hurricane-
harve&psig=AOvVaw1Yi59_AXqv6K0MgNrn5FB6&ust=1618941837032000&source=images&cd=vfe&ved=2ahUKEwjSt5G68orwAhV
617sIHShSDcYQjRx6BAgAEAc

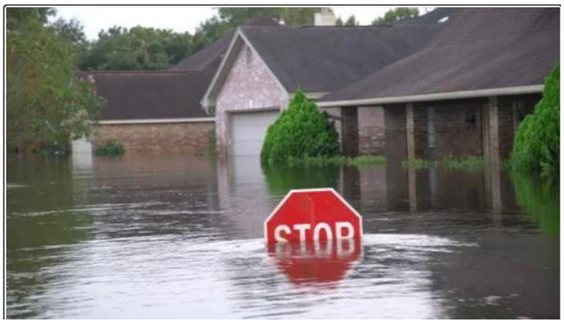

Mimage069

https://media.gettyimages.com/photos/street-signs-are-submerged-by-the-mississippi-river-june-18-2008-as-picture-
id81628070?s=612x612

https://www.google.com/url?sa=i&url=https%3A%2F%2Fwww.gettyimages.co.uk%2Fphotos%2Fmississippi-river-towns-brace-for-
major-flooding&psig=AOvVaw3aXnNkCZDKjd1ObYCqX3-Z&ust=1618941845993000&source=images&cd=vfe&ved=2ahUKEwjJsbS-
8orwAhX447sIHSk3AMkQjRx6BAgAEAc

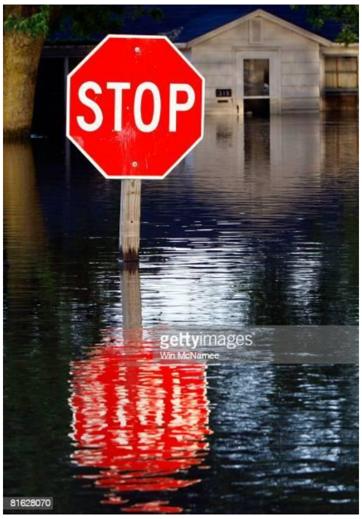

Mimage070

https://img1.wsimg.com/isteam/ip/79ff525d-d403-46e7-8840-eb6aed88632a/5ba9be50a367b.image.jpg

https://www.google.com/url?sa=i&url=https%3A%2F%2Fcaseconsultantsinternational.com%2Fcommunity-service%2Ff%2Fhow-to-
contribute-to-horry-county-flood-resilience-plan&psig=AOvVaw0j-A8ROig87YN-
JcxYYJfW&ust=1618937860025000&source=images&cd=vfe&ved=2ahUKEwiNnODR44rwAhWN6rsIHerdArcQjRx6BAgAEAc

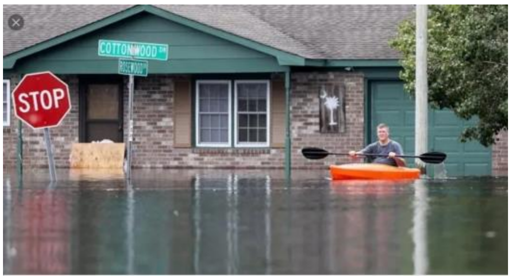

Mimage071

https://media.tegna-media.com/assets/KFSM/images/6c4e7a5d-df05-4031-bb0f-de1c3a81af21/6c4e7a5d-df05-4031-bb0f-de1c3a81af21_1920x1080.jpg

https://www.google.com/url?sa=i&url=https%3A%2F%2Fwww.5newsonline.com%2Fvideo%2Fweather%2Fgardenwalk-apartments-in-alma-evacuated-due-to-flooding%2F527-e0076637-4ad0-45e6-8940-e914d80031f5&psig=AOvVaw2nayYAusOCR8KGUTYwOg57&ust=1618937861606000&source=images&cd=vfe&ved=2ahUKEwjv4MDS44rwAhVN2rsIHWAXB4EQjRx6BAgAEAc

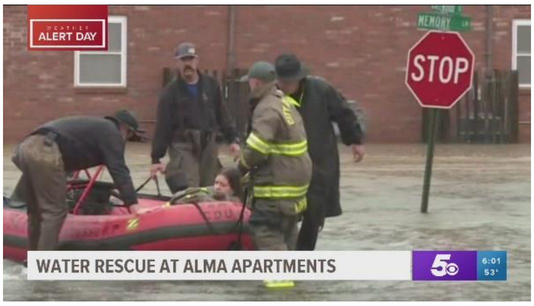

Mimage072

https://www.weathernationtv.com/app/uploads/2020/06/cover.jpg

https://www.google.com/url?sa=i&url=https%3A%2F%2Fwww.weathernationtv.com%2Fnews%2Fmississippi-valley-severe-storms-heavy-rain-from-cristobal%2F&psig=AOvVaw0is9kOWqILdPNRdvTJm27Y&ust=1618936184389000&source=images&cd=vfe&ved=2ahUKEwimvd-y3YrwAhVb2rsIHTM1DCoQjRx6BAgAEAc

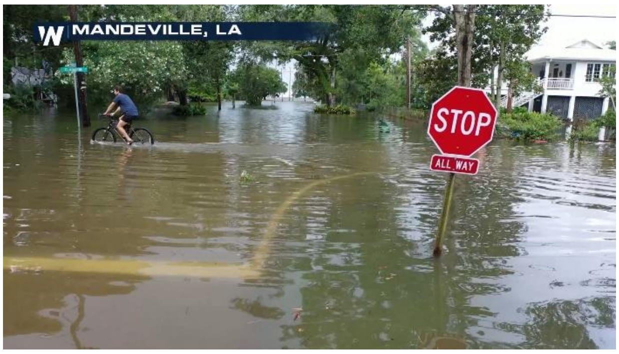

Mimage073

https://www.usnews.com/dims4/USNEWS/e4d0e40/2147483647/thumbnail/640x420/quality/85/?url=http%3A%2F%2Fmedia.beam.usnews.com%2F7f%2F5b88368f7a410bb1a1de6c64a5d9a2%2Fmedia%3Ab55750ba4a7147ad952708275a9de0f2SevereWeather.JPEG

https://www.google.com/url?sa=i&url=https%3A%2F%2Fwww.usnews.com%2Fopinion%2Feconomic-intelligence%2F2015%2F06%2F11%2Ftexas-flood-will-test-obama-disaster-relief-order&psig=AOvVaw2rPKCmgHrrjFBfRe31P5x_&ust=1618666665898000&source=images&cd=vfe&ved=2ahUKEwj2oKiu8YLwAhWVh_0HHfo2CjEQjRx6BAgAEAc

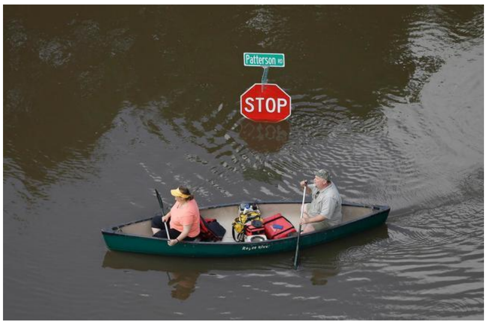

Mimage074video

https://ak.picdn.net/shutterstock/videos/26861191/thumb/1.jpg

https://www.google.com/url?sa=i&url=https%3A%2F%2Fwww.shutterstock.com%2Fvideo%2Fclip-26861191-stop-sign-pokes-out-muddy-river-flowing&psig=AOvVaw1m65glcRpxMiUnY71p02x6&ust=1618666667697000&source=images&cd=vfe&ved=2ahUKEwiciZav8YLwAhUx5bsIHZBwAGoQjRx6BAgAEAc

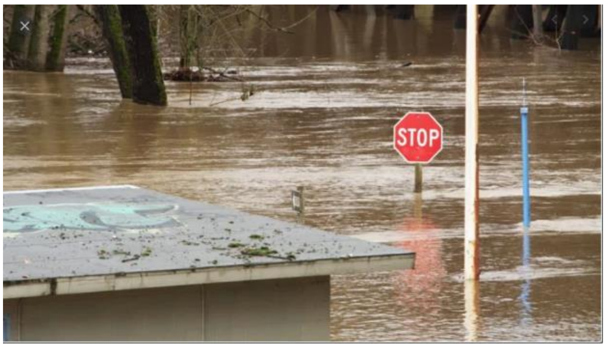

Mimage075

https://i.guim.co.uk/img/media/c4e79853c6076db9f77f8209164197771dc6f775/0_27_3500_2102/master/3500.jpg?width=1200&height=1200&quality=85&auto=format&fit=crop&s=2f7c2f811f60a988c6211751949044de

https://www.google.com/url?sa=i&url=https%3A%2F%2Fwww.theguardian.com%2Fnews%2F2020%2Fjan%2F31%2Fweatherwatch-how-repeated-flooding-can-shift-levees&psig=AOvVaw0RcK4Gwm0nl7iLetjrTP-y&ust=1618666741412000&source=images&cd=vfe&ved=2ahUKEwj6rKnS8YLwAhUX4bsIHRhZB_cQjRx6BAgAEAc

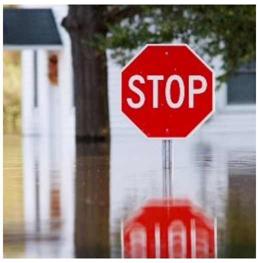

Mimage076

https://www.wave3.com/resizer/LUJiCz1gO_Xw3wYyGEXbTd4TwAs=/1200x0/arc-anglerfish-arc2-prod-raycom.s3.amazonaws.com/public/35WHMB4R2JEAZGJ6K5CJ5DOUYQ.png

https://www.google.com/url?sa=i&url=https%3A%2F%2Fwww.wave3.com%2F2020%2F02%2F15%2Fupdate-more-road-closures-announced-due-flooding%2F&psig=AOvVaw3peij5re5Bugujm69ITO5y&ust=1618666795872000&source=images&cd=vfe&ved=2ahUKEwi6oaXs8YLwAhWzz7sIHclxDnsQjRx6BAgAEAc

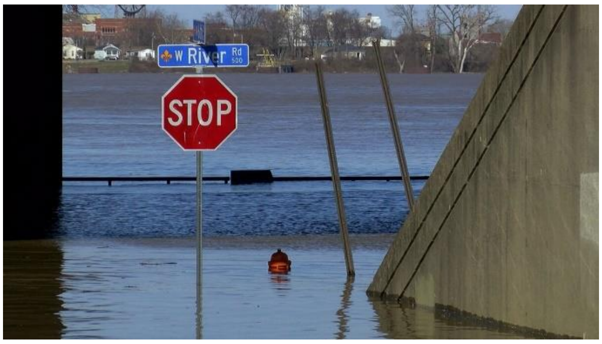

Mimage077

https://cdn.winknews.com/wp-content/uploads/2018/08/Capture391.jpg

https://www.google.com/url?sa=i&url=https%3A%2F%2Fwww.winknews.com%2F2020%2F06%2F29%2F15-million-u-s-homes-are-at-risk-of-flooding-50-higher-than-fema-estimates%2F&psig=AOvVaw0wgxg7AQAsdBUk7Q81SM-O&ust=1618666831245000&source=images&cd=vfe&ved=2ahUKEwipq5T98YLwAhW647sIHQc-DUQQjRx6BAgAEAc

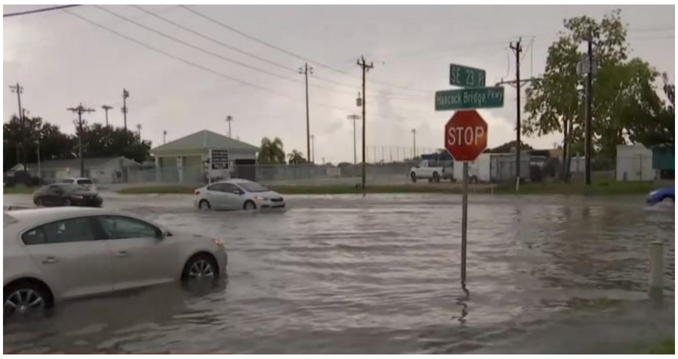

Mimage078

https://www.climate.gov/sites/default/files/styles/rotator_all/public/Missouri_flooding_2019_480.jpg?itok=saQx76CF

https://www.google.com/url?sa=i&url=https%3A%2F%2Fwww.climate.gov%2Ftags%2Fflooding&psig=AOvVaw0bvaLNcHUC3_Be3gS5hkc1&ust=1618666841913000&source=images&cd=vfe&ved=2ahUKEwjktZ-C8oLwAhXDVuUKHQbqA1AQjRx6BAgAEAc

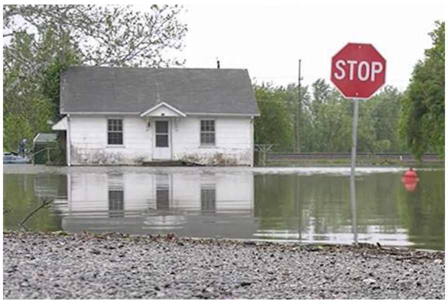
STOP
